# In Vitro and In Vivo Characterization of MCT1 Inhibitor AZD3965 Confirms Preclinical Safety Compatible with Breast Cancer Treatment

**DOI:** 10.3390/cancers13030569

**Published:** 2021-02-02

**Authors:** Zohra Benyahia, Marine C. N. M. Blackman, Loïc Hamelin, Luca X. Zampieri, Tania Capeloa, Marie L. Bedin, Thibaut Vazeille, Olivier Schakman, Pierre Sonveaux

**Affiliations:** 1 Pole of Pharmacology and Therapeutics, Institut de Recherche Expérimentale et Clinique (IREC), Université Catholique de Louvain (UCLouvain), Avenue Hippocrate 57 box B1.57.04, 1200 Brussels, Belgium; zohra.benyahia@uclouvain.be (Z.B.); marine.blackman@uclouvain.be (M.C.N.M.B.); loic.hamelin@uclouvain.be (L.H.); luca.zampieri@uclouvain.be (L.X.Z.); tania.demiranda@uclouvain.be (T.C.); marie.bedin@uclouvain.be (M.L.B.); thibaut.vazeille@uclouvain.be (T.V.); 2 Pole of Cell Physiology, Institut des Neurosciences (IoNS), Université Catholique de Louvain (UCLouvain), Avenue E. Mounier 53 box B1.53.17, 1200 Brussels, Belgium; olivier.schakman@uclouvain.be

**Keywords:** breast cancer, cancer metabolism, oxidative phosphorylation (OXPHOS), oxidative pathway of lactate, monocarboxylate transporters (MCTs), CD147/basigin, preclinical toxicology, muscle, heart, brain

## Abstract

**Simple Summary:**

The vast majority of tumors originate in tissues that use different substrates and oxygen to produce energy. However, tumors are disorganized structurally and functionally, which creates areas where oxygen and nutrients are poorly available. To survive and proliferate, cancer cells adapt by switching their metabolism to lactic fermentation. Their fate is further optimized by intercellular cooperation, but this creates a weakness that can be exploited therapeutically. Indeed, AZD3965 is a new drug currently tested in clinical trials that inhibits a cooperation based on lactate swapping for glucose between fermenting and respiring cells. It inhibits lactate transporter monocarboxylate transporter 1. Here, using malignant and nonmalignant cells representative of the breast tissue and several behavioral tests in mice, we establish that AZD3965 is safe for therapeutic use against cancer. The only side effect that we detected was a short-term memory retention defect that transiently perturbed the orientation of mice in space.

**Abstract:**

To survive and proliferate in solid tumors, cancer cells adapt and evolve rapidly in microenvironments where oxygen and substrate bioavailability fluctuates over time and space. This creates metabolic heterogeneity. Cancer cells can further cooperate metabolically, for example by swapping glycolytic end-product lactate for blood-borne glucose. This type of cooperation can be targeted therapeutically, since transmembrane lactate exchanges are facilitated by lactate-proton symporters of the monocarboxylate (MCT) family. Among new drugs, AZD3965 is a first-in-class selective MCT1 inhibitor currently tested in Phase I/II clinical trials for patients with different types of cancers. Because MCT1 can function bidirectionally, we tested here whether and how malignant and nonmalignant cells adapt their metabolism and MCT repertoire when AZD3965 inhibits either lactate import or export. Using breast-associated malignant and nonmalignant cell lines as models, we report that AZD3965 is not directly cytotoxic. In the presence of glucose and glutamine, oxidative cells can survive when lactate uptake is blocked, and proliferating cells compensate MCT1 inhibition by overexpressing MCT4, a specialized facilitator of lactate export. Phenotypic characterization of mice focusing on metabolism, muscle and brain physiology found partial and transient memory retention defect as sole consequence of MCT1 inhibition by AZD3965. We therefore conclude that AZD3965 is compatible with anticancer therapy.

## 1. Introduction

Oxidative phosphorylation (OXPHOS) is the main physiological mode of ATP production by adult mammalian cells, to the noticeable exception of erythrocytes that do not possess mitochondria. Depending on metabolic tissue preferences, several substrates (mainly glucose, lipids, glutamine and lactate) can fuel different catabolic pathways (glycolysis, lipolysis, glutaminolysis and the oxidative pathway of lactate) to aliment the tricarboxylic acid (TCA) cycle within mitochondria. The TCA cycle ultimately provides NADH and FADH2 that donate electrons to Complexes I and III of the electron transport chain (ETC), respectively. Electron motion from ETC Complex I to IV provides the proton motor force necessary for ATP production by Complex V/ATP-synthase. Importantly, maintenance of the electron flux requires enough oxygen to accept electrons released at Complex IV, yielding water as a final product.

Hypoxia, which can be biologically defined as a local level of oxygen below cellular needs, is a major characteristic of solid tumors [1]. It originates in microscopic tumors owing to the consumption of oxygen by layers of cancer cells progressively colonizing areas away from preexisting blood vessels. Hence, the threshold of oxygen diffusion across oxidative cell layers is estimated to range from 100 to 200 µm [2,3]. During further tumor evolution, additional tissue modifications aggravate hypoxia, which starts to exert its pervasive influence by fluctuating in time and space [4]. Cancer and host cells that fail to metabolically adapt die from necrosis. Conversely, those that adapt reversibly switch from an oxidative to a glycolytic metabolism (i.e., glycolysis starts to preferentially fuel lactate fermentation rather than the TCA cycle), survive and proliferate. Hypoxia-inducible transcription factors (HIFs) substantially participate in accelerating the glycolytic flux to maximize ATP production [5]. The counterpart of the glycolytic switch is an increased cellular dependency on glucose bioavailability, combined with extracellular matrix acidification largely due to lactic acid released through monocarboxylate transporters (MCTs) [6]. Importantly, these new activities resulting from adaptation to hypoxia may constitute druggable weaknesses for selective anticancer treatment.

Proliferation, another major characteristic of cancer cells, also benefits from a switch from an oxidative to a glycolytic metabolism. Indeed, the glycolytic pathway is a metabolic hub connected to biosynthetic pathways, such as the pentose phosphate pathway (PPP) and the serine pathway [7]. In the context of cell proliferation, switching from OXPHOS to aerobic glycolysis is controlled by growth factors and their (mutated) signaling pathways that accelerate the glycolytic flux. When glycolysis becomes the main mode of energy production, mitochondria are greatly relieved from their bioenergetic task and may contribute to the biosynthesis of cell components, by using reductive glutaminolysis to fuel lipogenesis for example [8]. Such metabolic rewiring is known as the Warburg Effect. Warburgian cancer cells thus alternate periods of glycolytic energy production and biosynthesis along the cell cycle [9].

Collectively, tumors thus comprise oxidative cancer cells in normoxic areas, glycolytic cancer cells in hypoxic areas and Warburgian cancer cells disseminated across the entire lesion. In clinical tumors, the latter generally constitutes a minority, with Ki67 nuclear staining in human tumors showing on average ~20–25% positivity in breast cancers [10], ~17% in colorectal cancers [11] and ~3–5% in prostate cancers [12]. It is therefore incorrect to consider Warburgian cancer cells performing aerobic glycolysis as the main cancer cell subpopulation in human tumors.

To optimize the use of available resources, cancer cells with different metabolic activities can cooperate. In 2008, we [13] identified a cooperation based on the intercellular exchange of lactate, where glycolytic cancer cells produce and release lactate and oxidative cancer cells take up and oxidize lactate, thus sparing glucose for hypoxic cancer cells. In the cooperation that we view as a symbiosis, hypoxic cancer cells with a strict dependency on glucose for ATP production gain access to more glucose, and oxidative cancer cells are rewarded by increased autophagy, which facilitates the recycling of oxidized cell components [14].

Swapping lactate for glucose depends on the expression and activity at the cell plasma membrane of lactate-proton symporters of the MCT family (see references [15,16,17] for reviews). MCTs are passive and can thus function bidirectionally, depending on the gradients of lactate and protons across the plasma membrane. Among them, MCT4 is well adapted to export lactate; although it has a low Km for lactate (Km = 22–28 mmol/L), it is hypoxia-inducible and has a high turnover rate [18,19]. Conversely, MCT1 has a higher affinity for lactate (Km = 3.5–10 mmol/L) and can function either to import or to export lactate when expressed by oxidative or glycolytic cells, respectively [16]. Among other MCTs, MCT2 has the highest affinity for lactate (Km = 0.5–0.75 mmol/L) and has been reported to be expressed in prostate cancer [20], whereas MCT3 expression is restricted to the retinal pigments and choroid plexus epithelium of the eyes [21].

With the discovery of the lactate-based metabolic symbiosis of tumors, we directly envisioned MCTs as potential anticancer targets [13]. Our preference goes for MCT1 because (i) it is expressed in oxidative cancer cells located at the vicinity of functional blood vessels delivering systemic treatments, and (ii) as a target it would offer anticancer selectivity by corrupting the metabolic symbiosis between glycolytic and oxidative cancer cells. Accordingly, upon MCT1 inhibition we observed that oxidative cancer cells switched substrate from lactate to glucose and survived, which indirectly killed hypoxic cancer cells through glucose starvation in experimental tumors [13]. In comparison, targeting MCT4 would directly kill glycolytic cells, malignant or not, by virtue of intracellular acidification. Similarly, inhibiting MCT1 in glycolytic cells could kill malignant and nonmalignant cells that use MCT1 to export lactate, unless they would compensate by increasing MCT4 expression or by switching to a more oxidative metabolism, as previously reported in cancer cells [22,23].

With a ~10-fold selectivity for MCT1 (Ki = 1.6 nmol/L) compared to MCT2 (Ki = 20 nmol/L) and no inhibitory activity on MCT4 [24], AZD3965 entered Phase I/II clinical trials in 2013 in UK for patients with a solid tumor, diffuse large B cell lymphoma or Burkitt lymphoma (clinicaltrials.gov NCT01791595). AZD3965 binds MCT1 key residues Lys38, Asp302 and Arg306 involved in lactate transport within transmembrane helixes 7–10, and thus inhibits MCT1 transporter activity [25]. The selectivity of AZD3965 for MCT1 compared to MCT2 is further due to an interaction of MCT1 with chaperone protein CD147/basigin, which facilitates drug access and/or binding [26], whereas MCT2 preferentially interacts with gp70/embigin [27].

AZD3965 is a promising new anticancer agent. However, cancers are not the sole entities to exchange lactate. Physiologically, skeletal muscles and the brain are, indeed, privileged sites of oxidative lactate metabolism. In muscles, force-producing fast-twitching muscle fibers indeed become quite rapidly glycolytic (i.e., depending on exercise strength, duration and training). They start to release lactate through MCT4, which is recaptured and oxidized as a mitochondrial fuel by slow-twitching muscle fibers expressing MCT1 [28]. In the brain, astrocytes fuel neurons with lactate, which depends on MCT1 and MCT4 for lactate export by astrocytes and on MCT2 for lactate uptake by neurons [29]. Neurons then use lactate oxidatively. Overall, when considering the whole body, lactate has been described as an oxidative fuel at least as important as glucose [30], leaving open the possibility of life-threatening side effects arising from MCT1 inhibition, including with the use of AZD3965.

Here, we hypothesized that oxidative cells using MCT1 to import lactate could survive to MCT1 inhibition by either switching substrate and/or by increasing the expression of another MCT isoform, especially MCT2. Conversely, glycolytic cells should display lesser metabolic plasticity, and were expected to primarily compensate MCT1 inhibition by upregulating another MCT isoform, in particular MCT4. This double hypothesis was tested in human cell lines representative of the breast tissue, whether human breast cancer cells or human breast-associated nonmalignant cells. They were submitted to different culture conditions and treated ± AZD3965. To fill a gap in the existing scientific literature, we further aimed to characterize the neuromuscular consequences of MCT1 inhibition by AZD3965 on mouse physiology. Others previously demonstrated the strong anticancer effects of AZD3965 in preclinical models of breast cancer [31]. Our ultimate goal was thus to establish the preclinical safety profile of MCT1 inhibition by AZD3965.

## 2. Results

### 2.1. Model Selection Based on the Characterization of MCT1 and CD147 Expression

To test the consequences of MCT1 inhibition by AZD3965 on cancer and nonmalignant cells representative of the breast tissue, we chose as models T47D human ductal carcinoma breast cancer cells, MCF7 human breast adenocarcimoma cancer cells, nonmalignant MCF10A human epithelial breast cells and human BJ fibroblasts. These cell lines were selected because they all expressed MCT1 at mRNA (Figure S1A) and protein (Figure S1B) levels in basal culture conditions, and the transporter was located at the plasma membrane together with CD147/basigin (Figure S1C).

**Figure 1:**
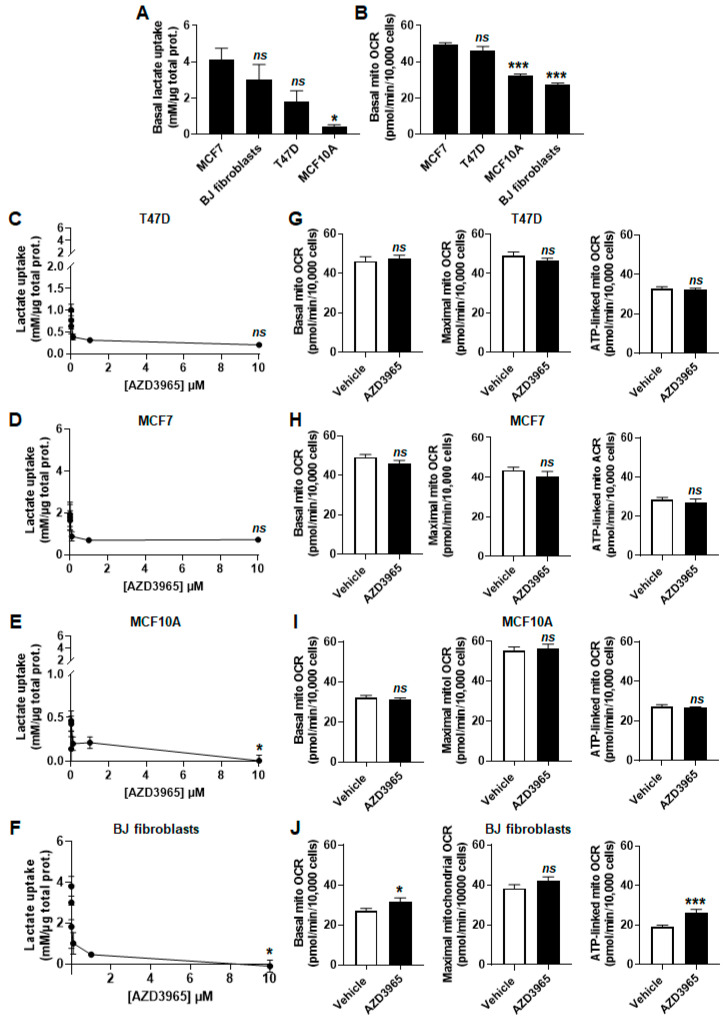
Malignant and nonmalignant cells representative of the breast consume lactate. (**A**–**J**) Cells were metabolically characterized in medium containing 10 mmol/L sodium *L*-lactate, no glucose, no glutamine and 1% FBS. (**A**) Lactate uptake over 24 h by untreated MCF7 and T47D human breast cancer cells, BJ normal human fibroblasts and MCF10A nonmalignant human breast epithelial cells (*n* = 3–9). (**B**) Basal mitochondrial oxygen consumption rate (mito OCR) of untreated MCF7, T47D, MCF10A cells and BJ fibroblasts (*n* = 11–22). (**C**) Lactate uptake over 24 h by T47D cells exposed to increasing concentrations of MCT1 inhibitor AZD3965 (*n* = 3). (**D**) As in C, but using MCF7 cells (*n* = 8–9). (**E**) As in C, but using MCF10A cells (*n* = 3). (**F**) As in C, but using BJ fibroblasts (*n* = 3). (**G**) Basal mitochondrial oxygen consumption rate (mito OCR; left panel), maximal mito OCR (middle panel) and mito OCR linked to ATP production (right panel) of T47D cells treated for 24 h ± 10 µmol/L AZD3965 (*n* = 9–12). (**H**) As in G, but using MCF7 cells (*n* = 22–24). (**I**) As in G, but using MCF10A cells (*n* = 12). (**J**) As in G, but using BJ fibroblasts (*n* = 11). All data are shown as means ± SEM. * *p* < 0.05, *** *p* < 0.005, *ns p* > 0.05 compared to MCF7 (**A**–**B**) or to vehicle (**C**–**J**); by one-way ANOVA followed by Dunnett post-hoc test (**A**–**F**) or Student’s *t* test (**G**–**J**).

To test MCT1 as an inward lactate transporter, cells were assayed in medium containing 10 mmol/L of sodium *L*-lactate (corresponding to the average concentration of lactate in human tumors) [32], no glucose, no glutamine and 1% fetal bovine serum (FBS). Enzymatic measurements of extracellular lactate levels revealed that all cell lines had the capacity to import lactate (Figure 1A). T47D, MCF7 cells and BJ fibroblasts were equally efficient, whereas MCF10A cells consumed significantly less lactate than MCF7 cells over 24 h. In the same experimental conditions, oxygen consumption rate (OCR) measurements further established that lactate fueled the mitochondrial respiration of all cell lines, although more efficiently in malignant than in nonmalignant cells (Figure 1B).

In addition to MCT1, all cell lines also expressed MCT2 at mRNA level (Figure S1A; where *SLC16A7/MCT2* is barely detectable in T47D, MCF10A cells and BJ fibroblasts), as well as at protein level (Figure S1B). MCT2 was present at the plasma membrane of MCF10A cells and BJ fibroblasts, but was mostly cytosolic in T47D (where it was barely detectable) and MCF7 cancer cells (Figure S1C). MCT4 was also expressed by all cell lines with the lowest relative protein expression in cancer cells (Figure S1A,B). It was located at the plasma membrane only in MCF10A cells (Figure S1C). The chaperone protein CD147/basigin shared by both MCT1 and MCT4 [27] was present at the plasma membrane of all cell types (Figure S1C).

### 2.2. MCT1 Is the Main Facilitator of Lactate Uptake by Breast Cancer and Breast-Associated Nonmalignant Cells

When MCT1 was operating inwardly in the lactate assay medium, MCT1 inhibitor AZD3965 dose-dependently inhibited lactate uptake by all cell lines (Figure 1C–F). However, at the highest dose tested (10 µmol/L), inhibition was partial in cancer cells, with a residual uptake of lactate of 0.20 ± 0.01 mmol/L per µg of total proteins over 24 h by T47D cells (−65%; Figure 1C) and of 0.71 ± 0.06 mmol/L per µg total protein over 24 h for MCF7 cells (−72%; Figure 1D). In contrast, 10 µmol/L of AZD3965 completely inhibited lactate uptake by MCF10A cells (Figure 1E) and BJ fibroblasts (Figure 1F). Collectively, these experiments indicated that MCT1 is the main facilitator of lactate uptake in the four tested cell lines. Nevertheless, neither partial nor full inhibition of the transporter significantly repressed mitochondrial respiration (basal, maximal and ATP-linked mitochondrial OCRs) after 24 h of treatment (Figure 1G–J). Basal (+14%) and ATP-linked (+27%) mitochondrial OCRs even significantly increased in BJ fibroblasts (Figure 1J).

### 2.3. Inhibition of MCT1-Dependent Lactate Uptake by AZD3965 Does Not Kill Malignant and Nonmalignant Breast-Associated Cells

**Figure 2:**
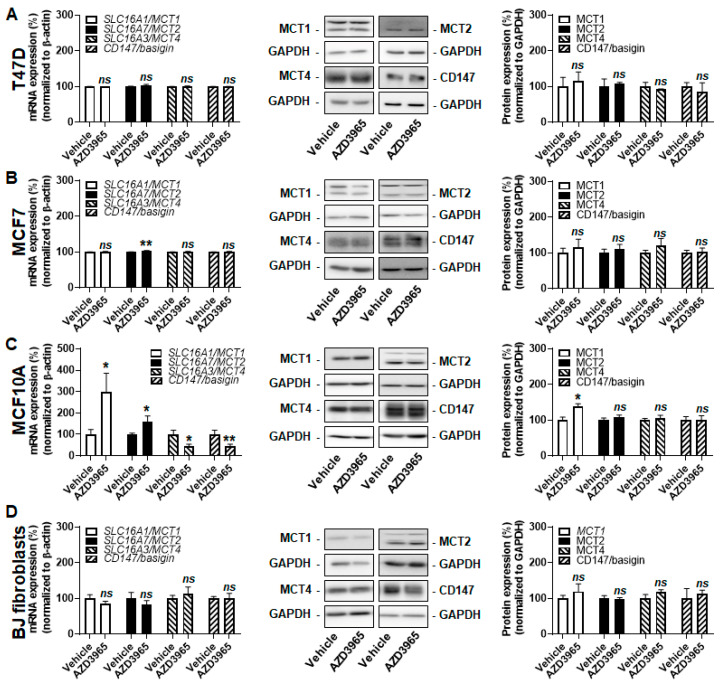
AZD3965 induces minor changes in the monocarboxylate (MCT) repertoire of breast-associated malignant and nonmalignant cells when lactate is used as an oxidative fuel. (**A**–**D**) Cells were assayed in medium containing 10 mmol/L sodium *L*-lactate, no glucose, no glutamine and 1% FBS. (**A**) mRNA (left panel) and protein (middle and right panels) expression of MCT1, MCT2, MCT4 and CD147/basigin in T47D cells treated for 24 h ± 10 µM AZD3965 (*n* = 3–9 for RT-qPCR, *n* = 3 for WB). (**B**) As in A, but using MCF7 cells (*n* = 9 for RT-qPCR, *n* = 3–11 for WB). (**C**) As in A, but using MCF10A cells (*n* = 9–26 for RT-qPCR, *n* = 3–6 for WB). (**D**) As in A, but using BJ fibroblasts (*n* = 8–21 for RT-qPCR, *n* = 3–6 for WB). All data are shown as means ± SEM. * *p* < 0.05, ** *p* < 0.01, *ns p* > 0.05 compared to vehicle; by Student’s *t* test (**A**–**D**).

Increased MCT4 expression was previously reported to compensate for MCT1 inhibition in cancer cells [24]. However, when lactate was the only available exogenous fuel, MCT1 inhibition using 10 µmol/L of AZD3965 during 24 h did not increase MCT4 transcription or protein expression (Figure 2A–D) in the tested cell lines. The expression of other MCT isoforms and of CD147/basigin stayed stable in T47D cells (Figure 2A) and in BJ fibroblasts (Figure 2D). Comparatively, there was a minor yet significant induction (+4%) of *SLC16A7/MCT2* transcription in MCF7 cells, which did not influence MCT2 protein expression (Figure 2B). MCF10A cells were more responsive, with induction of *SLC16A1/MCT1* and *SLC16A7/MCT2* and repression of *SLC16A3/MCT4* and *CD147/basigin* transcription (Figure 2C). Yet, only MCT1 was induced (+39%) at the protein level. This response did not prevent full inhibition of lactate uptake by 10 µmol/L of AZD3965 (Figure 1E). We concluded that, when MCT1 functioned inwardly to facilitate lactate uptake, no changes in MCT or CD147/basigin expression functionally compensated AZD3965-induced MCT1 inhibition in our model cell lines.

**Figure 3:**
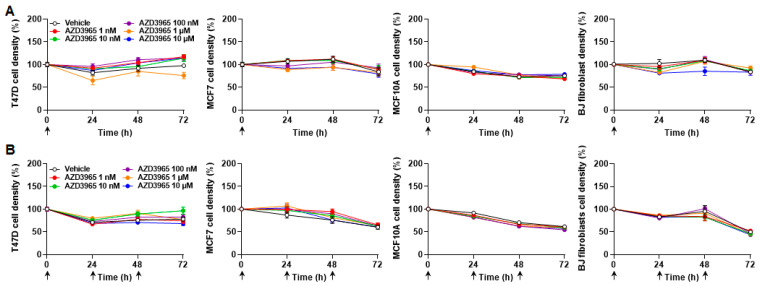
Lactate as an oxidative fuel ensures the survival of malignant but not of nonmalignant breast-associated cells, with no cytotoxic effect of AZD3965. (**A**,**B**) T47D, MCF7, MCF10A cells and BJ fibroblast density was assayed in medium containing 10 mmol/L sodium *L*-lactate, no glucose, no glutamine and 1% FBS. (**A**) Cell density over time after a single treatment at time 0 (arrow) with vehicle or different doses of AZD3965 (*n* = 8–16). (**B**) Cell density over time upon daily treatments (arrows) with vehicle or a given dose of AZD3965 (*n* = 15–16). All data are shown as means ± SEM. Analysis of cell death is presented in Table 1.

Interestingly, lactate alone was sufficient to ensure a 72 h survival of T47D and MCF7 cancer cells, but not of nonmalignant MCF10A epithelial cells nor BJ fibroblasts, which died at a very slow rate (Figure 3A and Table 1). Cancer cell proliferation was however halted. MCT1 inhibition by a single dose of AZD3965 (ranging from 1 nmol/L to 10 µmol/L) at time 0 did not significantly influence cancer cell survival and did not significantly accelerate cell death (Figure 3A and Table 1). In the same conditions, AZD3965 delivered daily for three consecutive days was not cytotoxic (Figure 3B and Table 1). At longer time points (up to 7 days), flow cytometry analyzes using annexin V and propidium iodide revealed that cells with lactate as only metabolic resource progressively died from necrosis (Figure S2). Apoptosis was marginal. AZD3965 (10 µmol/L) had no effects on apoptosis, but had a minor impact on necrosis, ranging from a limited yet significant increase for MCF7 cancer cells and BJ fibroblasts to a limited yet significant decrease for MCF7 cells (Figure S2). AZD3965 did not modulate the necrotic death of T47D cancer cells.

### 2.4. Inhibition of Lactate and Pyruvate Export by AZD3965 Induces an Oxidative Switch in Human Breast Cancer Cells, But Not in Human Breast-Associated Nonmalignant Cells

**Figure 4:**
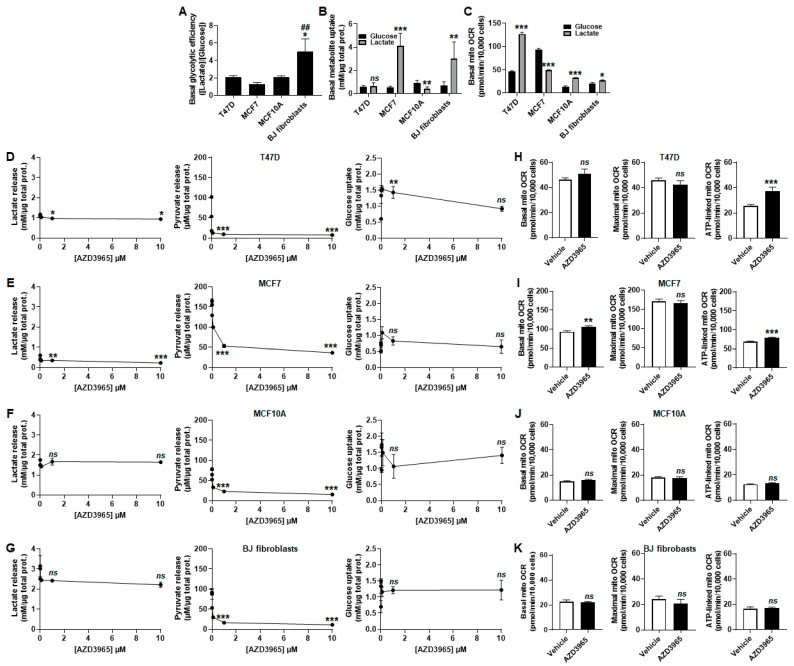
When MCT1 is used by proliferating cells to export lactate, AZD3965 induces an oxidative switch supporting ATP production in malignant but not in nonmalignant breast-associated cells. (**A**) Glycolytic efficiency of the cells cultured in medium containing 25 mmol/L glucose with 10 mmol/L GlutaMAX and 10% FBS, without added lactate, calculated as the lactate production/glucose consumption ratio (*n* = 3–5). (**B**) Glucose and lactate uptake over 24 h by untreated T47D, MCF7, MCF10A cells and BJ fibroblasts cultured in medium containing 25 mmol/L glucose with 10 mmol/L GlutaMAX and 10% FBS, without added lactate (to determine basal glucose uptake, black bars) or in medium containing 10 mmol/L sodium *L*-lactate with no glucose, no glutamine and with 1% FBS (to determine basal lactate uptake, grey bars) (*n* = 3–5). (**C**) Basal mito OCR of the cells cultured in medium containing 25 mmol/L glucose with 10 mmol/L GlutaMAX, 10% FBS and without added lactate (to determine glucose-dependent OCR, black bars) or in medium containing 10 mmol/L sodium *L*-lactate with no glucose, no glutamine and with 1% FBS (to determine lactate-dependent OCR, grey bars) (*n* = 11–22). (**D**–**K**) Cells were metabolically characterized in medium containing 25 mmol/L glucose with 10 mmol/L GlutaMAX, 10% FBS and without added lactate. (**D**) Lactate release (right panel), pyruvate release (middle panel) and glucose uptake (right panel) over 24 h by T47D cells treated with increasing doses of AZD3965 (*n* = 3–6). (**E**) As in D, but using MCF7 cells (*n* = 3–6). (**F**) As in D, but using MCF10A cells (*n* = 3–6). (**G**) As in D, but using BJ fibroblasts (*n* = 3). (**H**) Basal mito OCR (left panel), maximal mito OCR (middle panel) and mito OCR linked to ATP production (right panel) of T47D cells treated for 24 h ± 10 µM AZD3965 (*n* = 9–12). (**I**) As in H, but using MCF7 cells (*n* = 11). (**J**) As in H, but using MCF10A cells (*n* = 11). (**K**) As in H, but using BJ fibroblasts (*n* = 11–12). All data are shown as means ± SEM. * *p* < 0.05, ** *p* < 0.01, *** *p* < 0.005, *ns p* > 0.05 compared to T47D cells (**A**), to cells in glucose-containing medium (**B**,**C**) or to vehicle (**D**–**K**); ^##^ *p* < 0.01 compared to MCF7 cells; by one-way ANOVA followed by Dunnett’s post-hoc test (**A**,**D**–**G**) or Student’s *t* test (**B**,**C**,**H**–**K**).

In addition to its role as a metabolic substrate for OXPHOS, lactate is also well known as a glycolytic end-product. The importance of MCT1 to convey lactate export was tested by treating the breast model cell lines in proliferation phase with increasing concentrations of AZD3965 without added lactate, in assay medium containing 25 mmol/L glucose, GlutaMAX and 10% FBS. In the presence of glucose and glutamine, T47D, MCF7 and MCF10A cells were equally glycolytic, releasing about two molecules of lactate per molecule of glucose consumed, whereas BJ fibroblasts released significantly more lactate than what could be produced glycolytically (Figure 4A).

Comparing lactate and glucose as bioenergetic fuels revealed that MCF7 cells and BJ fibroblasts more proficiently imported lactate than glucose, whereas T47D cells imported equal amounts of the two substrates and MCF10A cells more proficiently imported glucose than lactate (Figure 4B). These results did not match with corresponding basal mitochondrial OCRs. Indeed, lactate more efficiently supported OXPHOS than glucose + GlutaMAX in T47D, MCF10A cells and in BJ fibroblasts, whereas the opposite was observed in MCF7 cancer cells (Figure 4C).

AZD3965 dose-dependently repressed lactate release by proliferating T47D and MCF7 cancer cells, but not by proliferating MF10A cells and BJ fibroblasts, all cultured in glucose- and GutaMAX-containing assay medium (Figure 4D–G left panels). When present, this inhibition was partial (−20% and −80% with 10 µmol/L of AZD3965 in T47D and MCF7 cells, respectively). Pyruvate release was near complete inhibition in all tested cell lines treated with 10 µmol/L of AZD3965 (Figure 4D–G middle panels). A transient increase in glucose uptake by T47D cells was noted, with no other significant changes in the rest of the cell lines (Figure 4D–G right panels). Upon MCT1 inhibition by 10 µmol/L of AZD3965, breast cancer cells increased their OXPHOS metabolism for ATP production (Figure 4H,I). Comparatively, nonmalignant cells did not (Figure 4J,K).

### 2.5. Inhibition of MCT1-Dependent Lactate Export by AZD3965 Does Not Kill Malignant and Nonmalignant Breast-Associated Cells When Glucose and Glutamine Are Available

**Figure 5:**
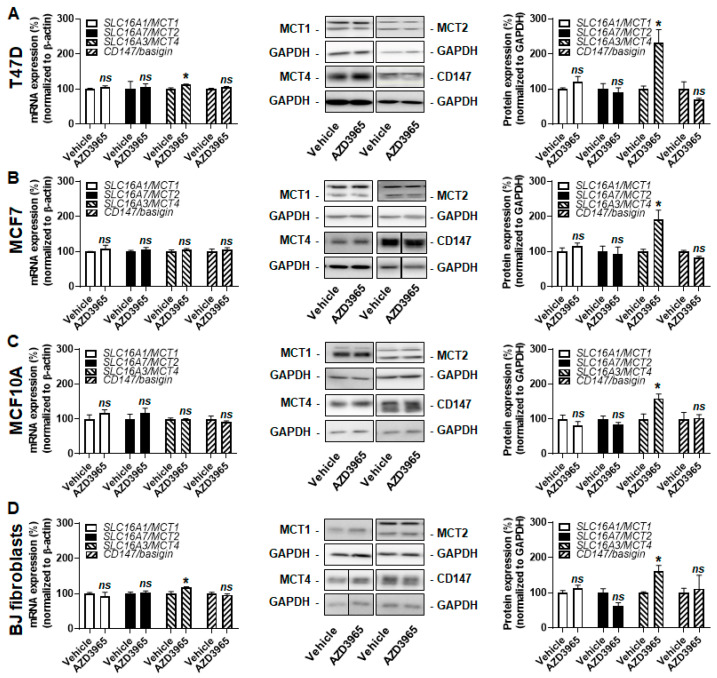
Proliferative breast-associated cells upregulate MCT4 expression upon MCT1 inhibition by AZD3965. (**A**–**D**) Cells were assayed in medium containing 25 mmol/L glucose with 10 mmol/L GlutaMAX, 10% FBS and without added lactate. (**A**) mRNA (left panel) and protein (middle and right panels) expression of MCT1, MCT2, MCT4 and CD147/basigin in T47D cells treated for 24 h ± 10 µM AZD3965 (*n* = 3–6 for RT-qPCR, *n* = 3 for WB). (**B**) As in A, but using MCF7 cells (*n* = 3–6 for RT-qPCR, *n* = 6 for WB). (**C**) As in A, but using MCF10A cells (*n* = 3–6 for RT-qPCR, *n* = 3–9 for WB). (**D**) As in A, but using BJ fibroblasts (*n* = 3–6 for RT-qPCR, *n* = 3–6 for WB). All data are shown as means ± SEM. * *p* < 0.05, *ns p* > 0.05 compared to vehicle; by Student’s *t* test (**A**–**D**).

At the molecular level, all tested cell lines treated with 10 µmol/L of AZD3965 increased MCT4 protein expression in a range of +59% to +131% (Figure 5A–D). Increased *SLC16A3/MCT4* transcription was detected in T47D (+13%) and BJ fibroblasts (+18%) (Figure 5A,D), but not in MCF7 and MCF10A cells (Figure 5B,C). The mRNA and protein expression of MCT1, MCT2 and CD147/basigin was unaltered.

**Figure 6:**
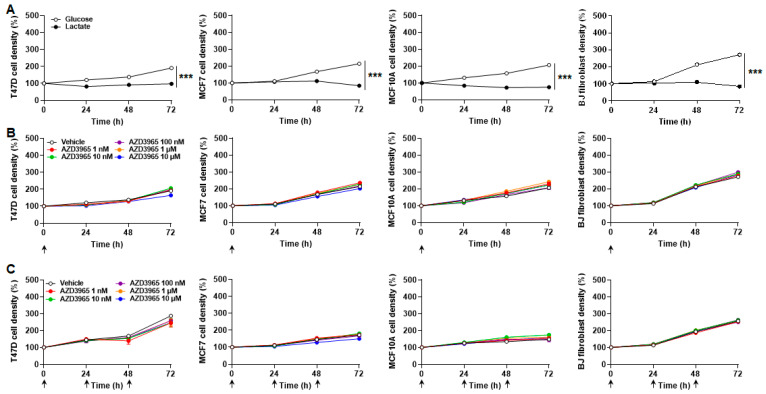
AZD3965 is not cytotoxic for proliferative breast-associated cells when glucose and glutamine are available as metabolic fuels. (**A**) Density of T47D, MCF7, MCF10A cells and BJ fibroblasts over time when cultured in medium containing 25 mmol/L glucose with 10 mmol/L GlutaMAX, 10% FBS and without added lactate (*white circles*) or in medium containing 10 mmol/L sodium *L*-lactate, no glucose, no glutamine and 1% FBS (black circles) (*n* = 8–16). (**B**,**C**) Cell density was assayed in medium containing 25 mmol/L glucose with 10 mmol/L GlutaMAX, 10% FBS and without added lactate. (**B**) Cell density over time after a single treatment (arrow) with vehicle or different doses of AZD3965 (*n* = 8). (**C**) Cell density over time upon daily treatments (arrows) with vehicle or a given dose of AZD3965 (*n* = 7–8). All data are shown as means ± SEM. *** *p* < 0.005 comparing whole curves using two-way ANOVA (**A**). Analysis of cell proliferation (**B**,**C**) is presented in Table 2.

Comparing the effects of bioenergetic fuels revealed that all cell types at best survived for short term with lactate as a sole fuel, but proliferated in the combined presence of glucose and glutamine (Figure 6A). Over 72 h, MCT1 inhibition by AZD3965 doses ranging from 1 nmol/L to 10 µmol/L did not repress cell proliferation when administered either as a single dose on time 0 (Figure 6B) or as three consecutive daily doses (Figure 6C).

### 2.6. AZD3965 Does Not Alter Muscle and Brain Physiology in Mice, to the Exception of Short-Term Memory Retention

**Figure 7:**
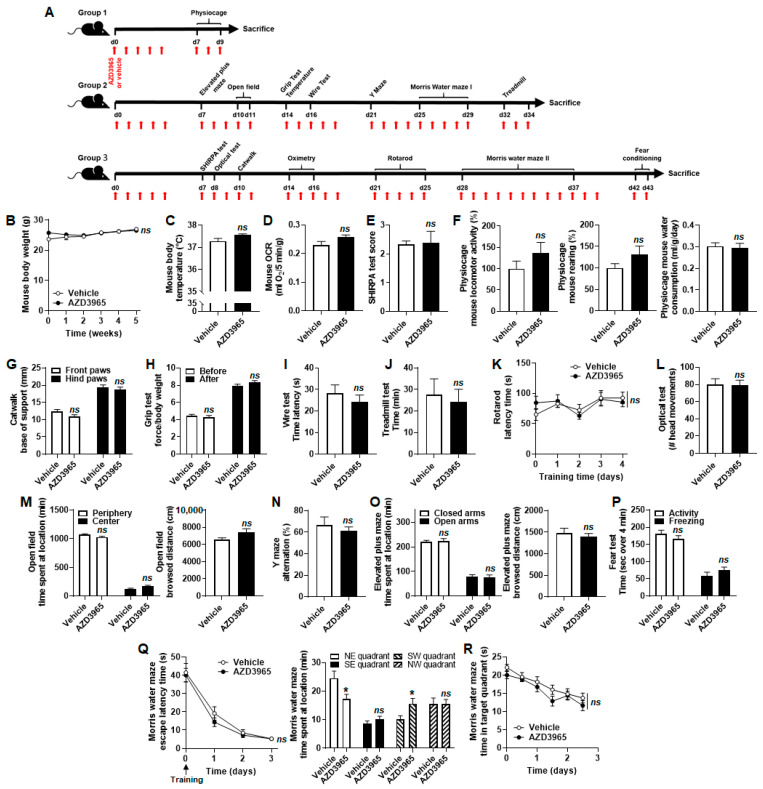
AZD3965 transiently reduces memory retention in mice, preserving other physiological metabolic, muscular and neuronal functions. (**A**) Cartoon depicting the sequence of phenotypic mouse experiments. Red arrows indicate treatment days (100 mg/Kg AZD3965 per os or vehicle). (**B**) Mouse body weight over time (*n* = 7–10). (**C**) Mouse body temperature after 15 days of treatment (*n* = 9). (**D**) Mouse OCR (*n* = 9–10). (**E**) Modified SmithKline Beecham, Harwell, Imperial College, Royal London Hospital, phenotype assessment SHIRPA test score (*n* = 9). (**F**) Mouse activity (left panel), rearing (middle panel) and water consumption (right panel) determined in physiocages (*n* = 5–6). (**G**) Base of support of the front paws (white columns) and hind paws (black column) in a catwalk assay (*n* = 7). (**H**) Grip test force normalized by mouse body weight (*n* = 8–11). (**I**) Time spent by mice hanging on a wire (*n* = 8–10). (**J**) Time spent by mice on a treadmill (*n* = 8–11). (**K**) Effect of daily training on the time spent by mice on a rotarod (*n* = 9–10). (**L**) Number of mouse head movements toward a rotating drum in the optical test (*n* = 8). (**M**) Exploration time (left graph) and browsed distance (right graph) of mice in open field (*n* = 8–11). (**N**) Path alternation on a Y maze (*n* = 8–11). (**O**) Time spent in closed and open arms (left graph) and distance browsed by mice (right graph) in an elevated plus maze (*n* = 8–11). (**P**) Activity and freezing times of the mice in a fear test (*n* = 9–10). (**Q**) Escape latency time (left graph) and time spent in each quadrant on day +3 (right graph) of a Morris water maze. Target quadrant was NE (*n* = 8–11). (**R**) Time spent by mice in the target NE quadrant in the absence of the platform (*n* = 9–10). All data are shown as means ± SEM. * *p* < 0.05, *ns p* > 0.05 compared to vehicle-treated animals; by two-way ANOVA testing the influence of treatments on whole curves (**B**,**K**,**Q** left,**R**) or Student’s *t* test (**C**–**J**,**L**–**P**,**Q** right).

AZD3965 has previously been shown to exert important anticancer effects, including in breast cancer-bearing mice [31]. However, little is currently known about the systemic toxicity of the drug. The experiments described above collectively suggested that nonmalignant cells were spared, so phenotypic assays in mice were conducted to conclude our study. AZD3965 was administered at a daily dose of 100 mg/kg per os with weekend breaks, as previously reported in preclinical pharmacokinetic [33,34] and therapeutic [24] studies. Our treatment schedule is depicted in Figure 7A. We focused on potential metabolic, muscular and neuronal toxicities because MCT1 gates lactate exchanges in skeletal muscles [28] and in the brain [29], especially.

Gross metabolic characterization first showed that mouse weight over time (Figure 7B), body temperature after 15 days of treatment (Figure 7C) and OCR (Figure 7D) were unaltered by AZD3965 compared to vehicle. The modified version of the SHIRPA test [35] was used for general phenotypic observation. Briefly, mice underwent 42 tests designed to observe a variety of spontaneous and provoked behaviors, which were scored [35]. AZD3965 had no significant impact (Figure 7E).

Potential influences of AZD3965 on mouse muscular function were first tested with voluntary exercise: locomotion, rearing and ability to drink were not modified (Figure 7F). We detected no change in gait (Figure 7G). Forced exercise consisted of a grip test that reports on the maximal muscular strength of mouse forelimbs. No difference was seen upon AZD3965 treatment compared to vehicle (Figure 7H). Similarly, we observed no difference in muscular fatigability in wire (Figure 7I) and treadmill (Figure 7J) tests. The latter assay as well as the rotarod test (Figure 7K) further revealed no loss of coordination or motor learning upon AZD3965 treatment.

Lastly, we tested neurological functions. AZD3965 did not affect mouse vision (Figure 7L) and exploration behaviors in open field (Figure 7M), Y maze (Figure 7N) and elevated plus maze (Figure 7O) assays. Fear perception was unaltered (Figure 7P). Spatial learning and memory were tested in a Morris water maze, where mice swimming in a circular pool of water virtually separated in four quadrants had to find an escape platform in the northeast (NE) quadrant. Vehicle and AZD3965-treated mice had similar escape times that similarly improved upon training (Figure 7Q, left). After 4 days of training, the escape platform was removed. On that day, AZD3965-treated mice spend less time (−30%) in the NE target quadrant and more time (+53%) in the opposite SW quadrant than vehicle-treated mice (Figure 7Q, right). It indicated a significant reduction of memory retention. Spatial memory extinction, however, was similar for AZD3965- and vehicle-treated mice (Figure 7R).

In control mice of Group 2 (Figure 7A), molecular analyses of tissues collected at the end of the experiments revealed similar relative expressions of *SLC16A1/MCT1*, *SLC16A7/MCT2* and *SLC16A3/MCT4* mRNAs in mouse skeletal muscle (gastrocnemius), heart and brain (Figure S3A). At the protein level, MCT2 was increasingly expressed from muscle to heart to brain to the detriment of MCT1, while MCT4 expression was essentially stable (Figure S3B). Comparing control to AZD3965-treated mice of Group 2, we detected no changes in the mRNA and protein levels of MCT1, MCT2, MCT4 and CD147/basigin (Figure S3C–E), to the exception of *SLC16A1/MCT1* mRNA expression that was significantly increased in mouse heart without consequence on MCT1 protein expression (Figure S3D). Conclusively, long-term (1 month) mouse treatment with AZD3965 did not affect the MCT repertoire of normal mouse skeletal muscle, heart and brain.

## 3. Discussion

This study first aimed to uncover how malignant and nonmalignant cells representative of the breast tissue adapt to AZD3965-induced MCT1 inhibition. It further aimed to establish the preclinical safety of this drug by conducting a battery of phenotypic tests in mice. Our main findings are that (i) up to a concentration of 10 µmol/L, AZD3965 is not directly cytotoxic to breast cancer cells and nonmalignant breast-associated cells, and (ii) AZD3965 tested at a therapeutic dose of 100 mg/Kg daily is safe in mice, with reversible, short-term memory retention defect as sole side effect identified.

MCT1 is an attractive anticancer target. As a conveyor of lactate across cell membranes, it not only gates lactate exchanges between glycolytic and oxidative cancer cells supporting a metabolic symbiosis [13], but also lactate entry into oxidative cancer cells and endothelial cells, which supports angiogenesis [36]. MCT1 also participates in tumor resistance to antiangiogenic therapies [37,38,39], and is critical for the cellular uptake of ketone bodies promoting mitochondrial biogenesis [40]. It is therefore not surprising that MCT1 inhibition exerts strong antitumor effects in many types of cancers, including breast cancer (see references [17,41] for detailed reviews).

With a Ki for MCT1 of 1.6 nmol/L, a Ki for MCT2 of 20.0 nmol/L and no inhibition of MCT3- or MCT4-mediated lactate transport [24], AZD3965 is a first-in-class MCT1 inhibitor resulting from almost three decades of research. Its selectivity for MCT1 over MCT2 is further enhanced by a preferred interaction of MCT1 with CD147/basigin, which may facilitate AZD3965 access and/or its binding to specific residues [26], whereas MCT2 preferentially interacts with gp70/embigin [27]. However, until now, little was known about the molecular responses of nonmalignant cells to AZD3965, and the scientific literature was largely silent concerning potential side effects of the drug.

In this study, we used malignant and nonmalignant cells representative of the breast tissue to test the hypothesis according to which cells would adapt to MCT1 inhibition. Breast was chosen because (i) human breast cancers generally have high MCT1 expression [31,42], (ii) AZD3965 was previously documented to exert strong anticancer effects in preclinical breast cancer models [31], (iii) AZD3965 has favorable pharmacokinetics in breast cancer-bearing mice with an intratumoral accumulation of ~1 µmol/L 12 h after the oral administration of 100 mg/Kg [33] and (iv) for the existence of a naturally immortalized nonmalignant human breast epithelial cell line (MCF10A) [43]. Even if they originate from the skin [44], we used BJ fibroblasts as a second nonmalignant cell line representative of the breast tissue for their long lifespan and because fibroblasts are particularly abundant in breast cancer [43].

When MCT1 was used as an inward transporter with lactate as only exogenous metabolic substrate, cells were forced to be oxidative, with basal OCRs equivalent to maximal OCRs in cancer cells, and basal OCRs lower than maximal oxidative capacities in nonmalignant cells. Cancer cells survived at least 72 h with lactate fueling mitochondrial ATP production, whereas nonmalignant cells slowly died (Table 1). This observation revealed that, compared to nonmalignant cells, cancer cells better solved the metabolic challenge imposed by an environment limited to lactate at its average concentration (10 mmol/L) in human tumors [45]. Yet, proliferation was halted. In this precise metabolic context, AZD3965 at a concentration of 10 µmol/L achieved full inhibition of lactate uptake by nonmalignant cells, but only partially inhibited lactate uptake by cancer cells. Apparently, the remaining ~30% of lactate uptake were sufficient to maintain cancer cell survival, which does not exclude the use of endogenous metabolic stores. Exhaustion of these resources would account for necrosis observed at later time points. In the absence of changes in the expression of MCT1, MCT2, MCT4 and CD147/basigin, residual lactate uptake could be explained by the relatively higher basal expression of MCT2 in cancer versus nonmalignant cells (Figure S1B). The involvement of other lactate transporters is unlikely, because MCF7 cells do not express the sodium-coupled monocarboxylate transporter SMCT1 [46] and, comparatively, a higher relative expression of MCT4 in nonmalignant cells did not compensate for lactate uptake inhibition by AZD3965. On the short term, nonmalignant cells died, but at a rate not different than upon vehicle treatment, confirming that lactate is not a suitable metabolic fuel for them. We previously drew the same conclusion for human endothelial cells that took up fewer lactate than necessary to maintain a sufficient OXPHOS rate [47]. On longer term (up to 7 days), necrosis was evidenced as the major form of cell death, which was only marginally impacted by AZD3965.

Unlike cancer cells, nonmalignant cells mounted metabolic and molecular responses to AZD3965-induced MCT1 inhibition. On the one hand, MCF10A epithelial cells upregulated MCT1 expression, but without any functional consequences on lactate uptake and cell survival. On the other hand, BJ fibroblasts accelerated the OCR linked to ATP production, hence OXPHOS. As lactate was the only exogenous substrate and its uptake was completely blocked, OXPHOS was necessarily fueled by intracellular components. They could be fatty acids, as BJ fibroblasts naturally store lipids that can be mobilized for lipolysis [48]. Activating autophagy would be a possible alternative. Nevertheless, their successful attempt to increase mitochondrial ATP production did not spare BJ fibroblasts from death, primarily caused by exogenous resources restricted to lactate.

To determine the consequences of AZD3965 delivery when MCT1 was functioning outwardly, we chose to challenge aerobic glycolysis (proliferating cells in a rich medium containing glucose, glutamine and serum) rather than anaerobic glycolysis (hypoxic cells). This choice was because hypoxia directly increases MCT4 expression through HIF-1 activation [18], which would have been a confounding factor for data interpretation. In the presence of glucose, glutamine and serum, all cell lines proliferated well and released lactate in a stoichiometric 2:1 ratio with glucose uptake. BJ fibroblasts were an exception, as they released more lactate that theoretically possible from glucose. It logically revealed that this cell line more than the others depends on a contribution of other oxidative substrate(s) for proliferation, thus producing additional lactate as previously shown with glutamine [49]. Overall, the Warburgian phenotype of all tested cell lines was associated with still largely preserved OXPHOS, demonstrating that lactic fermentation and OXPHOS are not mutually exclusive. All cell lines also released pyruvate in the ~100 µmol/L range, which corresponds to the physiological concentration of pyruvate in normal human blood [50]. At first glance, secreting pyruvate is an intriguing cell behavior. However, others proposed that pyruvate secretion is a mean for cancer cells to increase aerobic glycolysis [31]. Based on our observations, we extend this proposition to nonmalignant proliferating cells.

AZD3965 partially inhibited lactate export by malignant cells, but not by nonmalignant cells. All cell lines increased MCT4 expression. We interpret this change as a compensatory adaptation to MCT1 inhibition, based on the facts that (i) lactate release was systematically incompletely inhibited in our model cell lines, (ii) MCT4 is specialized in lactate export [19] and (iii) others previously suggested a same phenomenon, especially under hypoxia [22,23,51]. Of note, MCT4 transports lactate but not pyruvate [52], explaining why pyruvate export was always inhibited by AZD3965.

Increased MCT4 protein expression was sometimes but not systematically associated with increased *SLC16A3/MCT4* gene transcription. In our opinion, the main hypotheses to be tested are: (i) at the transcriptional level, a normoxic activation of HIF-1 by intracellular pyruvate (as previously reported for oxidative cancer cells and endothelial cells [36]), and (ii) postranscriptionally, MCT4 protein stabilization through increased interaction with CD147/basigin and/or reduced degradation kinetics.

Upon AZD3965 treatment, proliferating cancer cells, but not nonmalignant cells, accelerated OCR linked to ATP production, hence their OXPHOS rates. Such increase could primarily result from inhibition of pyruvate export [53], but discrepancies between cancer and nonmalignant cells rather indicate that selective lactate export inhibition in cancer cells is a main contributor to the switch, notwithstanding a possible contribution of other available oxidative substrates (glucose, glutamine, lipids).

Collectively, compensation of MCT1 inhibition by MCT4 and metabolic rewiring would explain why AZD3965 did not kill and barely altered the proliferation rate of breast-associated malignant and nonmalignant cells using MCT1 outwardly (Table 2).

These results prompted us to test the systemic consequences of AZD3965 administration. In preclinical mouse models, MCT1 inhibition is known to induce mild immunosuppression [54], which can be circumvented by encapsulating AZD3965 in pH-sensitive nanoparticles for preferential intratumoral release [55]. Other studies reported reduced visual acuity in rats (which resolved upon treatment cessation) [56] and aggravation of preexisting hyperlactemic acidosis in a human single case report [57]. Comparatively, disrupting MCT1 expression in the central nervous system is life-threatening [58]. The fact that AZD3965 entered and is still in Phase I/II clinical trials for cancer patients (clinicaltrials.gov NCT01791595) nevertheless indicates that the drug has acceptable side effects.

To clarify the situation, we administered AZD3965 orally to healthy mice at a dose of 100 mg/Kg daily with weekend breaks, i.e., slightly above the clinical trial regimen of 30 mg/Kg twice daily. None of the assays that tested animal metabolism, muscular and brain physiology unveiled any sign of AZD3965 toxicity, except the Morris water maze that evidenced a transient defect in memory retention (Figure 7Q,R). With a log*P* of 1.78 [59], AZD3965 is expected to cross the blood brain barrier. Our observation can thus be related to the occurrence of amnesia upon genetic *SLC16A1/MCT1* deletion in the brain of rats [60], with of course different scaling interpretation between incomplete pharmacological inhibition and gene silencing. Amnesia was not seen in our preclinical evaluation, as the learning curves of vehicle- and AZD3965-treated mice were similarly good (Figure 7Q), and spatial memory extinction followed similar kinetics (Figure 7R). Unlike in rats [56], we detected no optical symptoms in AZD3965-treated mice (Figure 7L). They would have affected several other behavioral assays, which was not the case. After one month of chronic MCT1 inhibition by AZD3965, mouse skeletal muscles, whole heart and whole brain retained unaltered MCT and CD147/basigin protein expression. We therefore concluded that AZD3965 administered at a dose exerting strong anticancer effects largely spared the functions of oxidative and glycolytic (skeletal muscles upon forced exercise) tissues in mice. The observed side effects are, in our opinion, compatible with anticancer treatment. They could be downscaled by further drug optimization aimed, for example, to modify blood–brain barrier crossing and to improve tumor selectivity and/or targeting.

## 4. Materials and Methods

### 4.1. Cells, Cell Culture and Treatments

T47D human ductal carcinoma breast cancer cells (catalogue #HTB-133), MCF7 human breast adenocarcimoma cancer cells (catalogue #HTB-22), nonmalignant epithelial MCF10A breast cells (catalogue #CRL-10317) and human BJ fibroblasts (catalogue #CRL-2522) were from the American type tissue collection (ATCC, Manassas, VA, USA). T47D and MCF7 cells are estrogen receptor-positive (ER+), progesterone receptor-positive (PR+) and epidermal growth factor receptor 2-negative (HER2-) [61]. They were routinely cultured in DMEM containing GlutaMAX (ThermoFisher, Erembodegem, Belgium; catalogue #61965), supplemented with 10 µg/mL insulin (Sigma-Aldrich, Overijse, Belgium) and 10% FBS (Sigma-Aldrich). BJ fibroblasts were routinely cultured in the same medium but without insulin. MCF10A cells were routinely cultured in DMEM/F12 containing GlutaMAX (ThermoFisher catalogue #31331093), supplemented with 10 mmol/L HEPES (ThermoFisher), 20 ng/mL epithelial growth factor (EGF; PeproTech, London, UK), 10 µg/mL insulin, 0.5 µg/mL hydrocortisone (Sigma-Aldrich) and 5% horse serum (ThermoFisher). All cells were grown in a humidified atmosphere at 37 °C, 5% CO_2_. Cell lines were successfully authenticated with a short tandem repeat (STR) test.

To test MCT1 as an inward lactate transporter, assay medium was DMEM containing no glucose and no glutamine (Sigma-Aldrich catalogue # D5030), supplemented with 10 mmol/L sodium *L*-lactate (Sigma-Aldrich) and 1% FBS. To test MCT1 as an outward lactate transporter, assay medium was DMEM containing 25 mmol/L glucose and 10 mmol/L GlutaMAX (ThermoFisher catalogue #61965), supplemented with 10% FBS. AZD3965 (5-[[(4S)-4-hydroxy-4-methyl-2-isoxazolidinyl]carbonyl]-3-methyl-1-(1-methylethyl)-6-[[5-methyl-3-(trifluoromethyl)-1H-pyrazol-4-yl]methyl]-thieno [2,3-d]pyrimidine-2,4(1H,3H)-dione)) from Selleckchem (Huissen, The Netherlands; catalogue #S7339) was prepared in DMSO and used at the indicated concentrations. An equal volume of DMSO (vehicle) served as control.

### 4.2. Lactate, Pyruvate and Glucose Measurements

Cells were seeded at 70,000 or 100,000 cells per well in 1 mL of assay medium. Lactate, pyruvate and glucose concentrations were measured in deproteinized cell supernatants using a CMA600 enzymatic analyzer (Aurora Borealis Control, Schoonebeek, The Netherlands), according to manufacturer’s instructions. All data were normalized to total protein content determined using the Bio-Rad Protein Assay (Temse, Belgium; catalogue # 5000006). The glycolytic ratio was calculated as lactate production/glucose consumption.

### 4.3. Cell Oximetry

Cell OCRs were determined using a Seahorse XF96 bioenergetic analyzer (Agilent, Diegem, Belgium) with the XF cell mito stress kit (Agilent) following manufacturer’s instructions. Briefly, 24 h before experiments, cells were plated at 10,000 or 20,000 cells per well in 96-well XF96 plates containing their respective routine culture media. On the day of analyses, media were replaced by assay media. Sequentially, basal OCR was acquired without treatment; ATP-linked OCR after the addition of 1 µmol/L of ATP synthase inhibitor oligomycin; maximal OCR after mitochondrial potential disruption using 1 µmol/L of ionophore carbonyl cyanide-4-(trifluoromethoxy)phenylhydrazone (FCCP); and non-mitochondrial OCR after the addition of 0.5 µmol/L of Complex I inhibitor rotenone together with 0.5 µmol/L of Complex III inhibitor antimycin A. Mitochondrial OCRs (mito OCRs) were calculated by subtracting non-mitochondrial OCRs to the corresponding basal, maximal and ATP-linked OCRs. All data were normalized to cell numbers at the end of experiments.

### 4.4. Reverse Transcription and Quantitative Polymerase Chain Reaction

For human cells, total mRNA was recovered as previously shown [62]. Reverse transcription (RT) followed by quantitative polymerase chain reaction (qPCR) on a Viia 7 system (ThermoFisher) were performed using a previously validated protocol [63]. *β-actin* mRNA expression served for data normalization. Primers were *SLC16A1/MCT1* sense 5′-GTT GGA CCC CAG AGG TTC TC-3′, antisense 5′-TGA GCC GAC CTA AAA GTG GTG-3′; *SLC16A7/MCT2* sense 5′-GCC AGA GAC CAG ATA AAG AGTC-3′, antisense 5′-GTC CCA GAG TCT TTG GTT CC-3′; *SLC16A3/MCT4* sense 5′-GTC ATC ACG GGG TTG GGT T-3′, antisense 5′-CGC TTG CTG AAG TAG CGG TT-3′; *CD147*/basigin sense 5′-TGC TGG TCT GCA AGT CAG AG-3′, antisense 5′-GCG AGG AAC TCA CGA AGA AC-3′; *β-actin* sense 5′-CCC GCG AGC ACA GAG C-3′, antisense 5′-TCA TCA TCC ATG GTG AGC TGG-3′.

For mouse tissues, whole brains and whole hearts were homogenized in the RA1/DTT buffer of the NucleoSpin RNA XS micro kit (Macherey-Nagel, Eupen, Belgium; catalogue #740902.50). Gastrocnemius muscles were homogenized in 1 mL of Tri reagent, with 3 to 5 repetitions of a Precellys Evolution homogenizer program consisting of 2 × 15 s at 5000 rpm with a 10 s pause. RT was conducted according to instructions of the RevertAid RT kit (ThermoFisher catalogue #K16910), with 1 μg of cDNA, 0.5 μL of random hexamer primers, 0.5 μL of oligo(dT) and a 90 min time of incubation at 42 °C. Two microliters of 1:5 diluted cDNA samples were used with 5 μL 2X Takyon low rox SYBR MasterMix dTTP Blue (Eurogentec, Liège, Belgium; catalogue #UF-LSMT-B0701), 0.2 μL of each primer (10 μM) and completed to 10 μL with water for qPCR analysis on a Viia 7 system (ThermoFisher). *β-actin* mRNA expression served for data normalization. Primers were *SLC16A1/MCT1* sense 5′- GCA GCA TGT GGT CCT CTC TTA AG -3′, antisense 5′- TGG TTC TCT TGT TAT CAG TGT TGG GTG -3′; *SLC16A7/MCT2* sense 5′- CTA CCA GGT TCT CCA GTG CTG T-3′, antisense 5′- ACG ACT GTT CCG CTG GCT ATG T-3′; *SLC16A3/MCT4* sense 5′- TCC ATC CTG CTG GCT ATG CTC T-3′, antisense 5′-CAG AAG GAC GCA GCC ACC ATT C -3′; *CD147*/basigin sense 5′-GAC ACT GGG GAA GAA GAG GC-3′, antisense 5′-GCA GTG AGA TGG TTT CCC GA-3′; *β-actin* sense 5′-GGC ACC ACA CCT TCT ACA ATG-3′, antisense 5′-GGG GTG TTG AAG GTC TCA AAC-3′.

### 4.5. Western Blotting and Immunocytochemistry

Western blotting (WB) on cell [64] and tissue [14] extracts was performed using previously disclosed protocols. All data were normalized to glyceraldehyde-3-phosphate dehydrogenase (GAPDH) expression, used as a loading control.

For WB on human cell extracts, primary antibodies were rabbit polyclonals for MCT1 (Bethyl Laboratories, Leiden, The Netherlands; catalogue #A304-358A) and MCT4 (Santa-Cruz, Heidelberg, Germany; catalogue #sc-50329); a goat polyclonal for MCT2 (GenWay Biotech, Huissen, The Netherlands; catalogue #GWB-78DDD0); a mouse monoclonal for CD147/basigin (BD Biosciences, Erembodegem, Belgium; catalogue #555961); and a rabbit monoclonal for GAPDH (Cell Signaling Technology, Leiden, The Netherlands; catalogue #2118). Secondary antibodies were horseradish peroxidase-conjugated goat anti-rabbit (Jackson ImmunoResearch, Huissen, The Netherlands; catalogue #111-035-003), goat anti-mouse (Jackson ImmunoResearch catalogue #115-035-003) and mouse anti-goat (Santa-Cruz #sc-2354). Images were captured on an Amersham 680 imager (GE healthcare, Diegem, Belgium). Note that CD147/basigin has different states of glycosylation and therefore appears as several bands on WB that were all quantified.

For WB on mouse tissue extracts, primary antibodies were rabbit polyclonals for MCT1 (Abcam, Cambridge, UK; catalogue #Ab93048), MCT2 (ThermoFisher catalogue #PA5-77498) and MCT4 (Millipore, Overijse, Belgium; catalogue #AB3314P); a goat polyclonal for CD147/basigin (R&D Systems, Abingdon, UK; catalogue #AF772); and a rabbit monoclonal for GAPDH (Cell Signaling Technology catalogue #2118). Secondary antibodies were the same as for WB on human cell extracts. Images were captured on an Amersham 680 imager (GE healthcare).

Immunocytochemistry (ICC) was also conducted as previously reported [13]. Primary antibodies for human cells were identical to those used for WB, except for MCT1 against which we used a rabbit polyclonal (Proteintech, Manchester, UK; catalogue #20139-1-AP). Secondary antibodies were Alexa Fluor 555 conjugate anti-rabbit (Cell signaling Technology catalogue #4413), Alexa Fluor 488 conjugate anti-mouse (Cell signaling Technology catalogue #4408) and Alexa Fluor 568 conjugate anti-goat (ThermoFisher catalogue #A11057). Cell nuclei were stained with 4′,6-diamidino-2-phenylindole dihydrochloride (DAPI). All uncropped WB images are shown in Figure S4.

### 4.6. Cell Density

On day 0, cells at a 70–80% confluence were treated as indicated. At the indicated time points, they were stained with crystal violet, as previously described [65]. Absorbances were measured at 570 nm on a SpectraMax miniMax 300 imaging cytometer (Molecular Devices, Munich, Germany).

### 4.7. Flow Cytometry

On day 0, cells were seeded at 40,000 to 100,000 cells per well in a 24 multiwell plate for 16 h, in DMEM containing no glucose and no glutamine (Sigma-Aldrich catalogue # D5030), supplemented with 10 mmol/L sodium *L*-lactate (Sigma-Aldrich) and 1% FBS. Cells were then treated ±10 µmol/L of AZD3965 and cultured up to 7 days. On each day, cell death was determined using the Annexin V Apoptosis Detection kit FITC (ThermoFisher catalogue #88-8005-74) according to manufacturer’s recommendations. Flow cytometric profiles were determined on a FACS AriaIII system (BD Biosciences). A minimum of 5000 events were acquired for each sample. Analyses were performed using the FlowJo software (BD Biosciences), where doublets were removed and single cells then categorized according to their Annexin V and propidium iodide status.

### 4.8. Mouse Experiments

All in vivo experiments were performed with approval (#2019/UCL/MD/027) of UCLouvain Comité d’Ethique pour l’Expérimentation Animale according to national and European animal care regulations. Eight weeks-old male C57BL/6JRj mice (Janvier, Le Genest-Saint-Isle, France) were randomly assigned to an experimental group undergoing sequential tests from the least to the most stressful, as depicted in Figure 7A. In each group, half of the mice received 100 mg/Kg of AZD3965 (MedChemExpress, Kampenhout, Belgium; Catalogue #HY-12750) per gavage 5 days per week until the end of experiments. The other mice received an equal amount of vehicle (DMSO 0.5%, methylcellulose 0.01%, Tween 80) using the same schedule. All tests are detailed in Appendix A. Mouse weight over 5 weeks of treatment and anal body temperature after 15 days of treatment were also measured. At the end of the experiments, mice were sacrificed by cervical dislocation, and organs (gastrocnemius muscles, whole heart and whole brain) were collected for mRNA and proteins expression analyses.

### 4.9. Statistics

All data are expressed as means ± standard error on the mean (SEM). Error bars are sometimes smaller than symbols. *n* refers to the total number of replicates per group. Data were analyzed using GraphPad Prism 8.4.3 (San Diego, CA, USA). Outliers were identified using Dixon’s Q test. Student’s *t* test, one sample *t* test, one-way ANOVA with Dunnett’s post-hoc test and two-way ANOVA were used where appropriate. *p* < 0.05 was considered to be statistically significant.

## 5. Conclusions

The limited direct cytotoxicity of MCT1 inhibitor AZD3965 for cells representative of the breast tissue is compatible with the symbiotic theory [13]. According to this theory applicable to tumors in vivo, targeted oxidative cancer cells would adapt metabolically, resulting in the indirect death of hypoxic/glycolytic cancer cells engaged in a dependent relationship with the former. In preclinical assays in mice, AZD3965 had limited side effects at a dose previously shown to be associated with strong anticancer effects. In addition to potential immune and optical side effects, ongoing and future clinical trials with AZD3965 should nevertheless pay special attention to the possibility of memory retention defects. Although mouse experiments cannot be directly translated to humans, basal MCT2 expression could be a potential cause of intrinsic treatment resistance, and increased MCT4 expression and/or OCR potential causes of acquired resistance to MCT1 inhibition.

## Figures and Tables

**Table 1:** Time (h) to reach 50% cell death in medium containing 10 mmol/L of sodium *L*-lactate, no glucose, no glutamine and 1% FBS ± AZD3965.

**[AZD3965], 1 dose, 72 h**							
	**Vehicle**	**1 nmol/L**	**10 nmol/L**	**100 nmol/L**	**1 µmol/L**	**10 µmol/L**	***n***
T47D	No cell death ^1^	No cell death	No cell death	No cell death	No cell death	No cell death	15–16
MCF7	No cell death	No cell death	No cell death	No cell death	No cell death	No cell death	15–16
MCF10A	194 ± 20	152 ± 15	171 ± 19	241 ± 45	165 ± 21	222 ± 26	15–16
BJ fibro	460 ± 96	299 ± 34	396 ± 130	283 ± 25	No cell death	141 ±36	15–16
**[AZD3965], 3 daily doses, 72 h**							
	**Vehicle**	**1 nmol/L**	**10 nmol/L**	**100 nmol/L**	**1 µmol/L**	**10 µmol/L**	***n***
T47D	181 ± 31	No cell death	No cell death	No cell death	187 ± 42	138 ±51	15–16
MCF7	103 ± 15	No cell death	138 ± 25	153 ± 30	68 ± 35	106 ± 8	15–16
MCF10A	119 ± 13	120 ± 14	109 ± 11	88 ± 7	104 ± 10	95 ± 7	16
BJ fibro	73 ± 3	82 ± 7	66 ± 4	68 ± 6	63 ± 2	59 ± 1	15–16

^1^ Significant cell death was determined with a one-sample t test compared to steady state. Effects of AZD3965 compared to vehicle were tested using one-way ANOVA with Dunnett’s post-hoc test. All *p*-values are >0.05.

**Table 2:** Cell doubling times (h) in medium containing 25 mmol/L glucose, 10 mmol/L GlutaMAX and 10% FBS ± AZD3965.

**[AZD3965], 1 dose, 72 h**							
	**Vehicle**	**1 nmol/L**	**10 nmol/L**	**100 nmol/L**	**1 µmol/L**	**10 µmol/L**	***n***
T47D	78 ± 3	77 ± 3	71 ± 4	75 ± 5	74 ± 4	103 ± 5 ***	8
MCF7	62 ± 3	58 ± 2	57 ± 1	64 ± 1	62 ± 2	72 ± 3 *	7–8
MCF10A	70 ± 3	60 ± 1	63 ± 2	72 ± 6	57 ± 2 *	63 ± 3	8
BJ fibroblasts	50 ± 1	49 ± 1	47 ± 1	46 ± 1 *	47 ± 1	48 ± 1	8
**[AZD3965], 3 daily doses, 72 h**							
	**Vehicle**	**1 nmol/L**	**10 nmol/L**	**100 nmol/L**	**1 µmol/L**	**10 µmol/L**	***n***
T47D	49 ± 5	52 ± 6	52 ± 2	53 ± 3	51 ± 4	59 ± 7	7–8
MCF7	87 ± 6	98 ± 11	86 ± 4	100 ± 4	88 ± 4	129 ± 4 **	7–8
MCF10A	132 ± 15	127 ± 16	91 ± 3	164 ± 24	108 ± 8	123 ± 13	7–8
BJ fibroblasts	53 ± 1	54 ± 1	51 ± 0.5	52 ± 0.5	53 ± 0.5	55 ± 1	7–8

* *p* < 0.05, ** *p* < 0.01, *** *p* < 0.005 compared to vehicle; by one-way ANOVA with Dunnett’s post-hoc test.

## Data Availability

Data are contained within the article or Supplementary Material.

## Supplementary Materials

The following are available online at https://www.mdpi.com/2072-6694/13/3/569/s1, Figure S1: MCTs and CD147/basigin expression in breast-associated cells, Figure S2: Long-term culture with lactate as only exogenous resource induces breast-associated cell necrosis with limited impact of additional MCT1 inhibition by AZD3965, Figure S3: A chronic treatment with AZD3965 does not alter the expression of MCTs and CD147/basigin in mouse skeletal muscles, heart and brain, Figure S4: Uncropped WB images.

## Appendix A. Detailed Materials and Methods of Mouse Experiments

### Appendix A.1. Mouse Oximetry

The OCR of mice was measured in a homemade metabolic chamber maintained at room temperature. The system consisted of a cylindrical plastic chamber equipped with a removable cage containing water-saturated soda lime (Medisorb). Each mouse was sealed in the chamber, and variations of the internal pressure directly proportional to oxygen consumption were measured by a nanometer. A syringe was used to deliver the volume of air required. Data were recorded for 5 min after a 15 min acclimatization period, and normalized for mouse weights.

### Appendix A.2. Modified SHIRPA Test

The modified version of the SHIRPA test [35] was used for gross phenotypic observation. Mice underwent 42 tests aimed to observe a variety of spontaneous and provoked behaviors. The European Mouse Phenotyping Resource for Standardized Screens (EMPReSS) score sheet was used to record the observations.

### Appendix A.3. Physiocage Assay

Basal mouse locomotor activity and rearing as well as water intake were measured in individual physiocages (Bioseb, Vitrolles, France) for 48 h after 24 h of acclimatization, according to manufacturer’s instructions.

### Appendix A.4. Catwalk

Catwalk was used to test gait during voluntary locomotion. In a Catwalk 7 system (Noldus, Wageningen, The Netherlands), paw prints were recorded, allowing the analysis of the distance between front paws and between hind paws. Five runs were recorded per mouse, and analyses were performed on the fastest uninterrupted run.

### Appendix A.5. Grip Test

The grip test was used to measure the force of the forelimb muscles. In an animal grip strength system (Bioseb), mice gripped a metal mesh grid with both fore paws and were gently pulled in direction of their tails, using a slow horizontal motion. The grid was connected to a digital gauge recording the peak force in grams. Two consecutive measurements were taken for each animal, and means of the highest values of force recorded were normalized to mouse body weights and used for analyses.

### Appendix A.6. Wire Test

The wire test was used to assess muscular fatigability. Three consecutive times with 30 s recovery periods, mice were suspended by their forelimbs to a 1.5 mm-thick, 60 cm-long metallic wire 45 cm above soft ground. Mean times to fall (latency times) were used for analyses, with a maximal time of 180 s (end of the experiment).

### Appendix A.7. Treadmill Test

A treadmill was used to measure muscular fatigability upon forced exercise. Mice were placed on a homemade treadmill with an inclination of 15° and an initial speed of 6 m/min, which was increased by 1 m/min every minute up to 18 m/min. The back of the treadmill was equipped with a grid that discharged a mild electrical current as a stimulus aimed at motivating the animal to keep running on the treadmill. The time to exhaustion was measured for each mouse, with a maximal time of 60 min (end of the experiment).

### Appendix A.8. Rotarod Test

The rotarod test was used to assess motor skills (grip, balance, coordination). Every day for 4 consecutive days, mice were placed 3 times per day with 10 min breaks on a rotating rod (Bioseb) under continuous acceleration from 4 to 40 rpm every 8 s. The mean time spent by each mouse on the rod before falling off (latency time) was recorded, with a maximal duration of the experiment of 300 s.

### Appendix A.9. Optical Test

Mice were placed on a 9.5 cm-diameter round platform surrounded by a 30 cm-diameter motorized drum rotating at 2 rpm. After 10 min of adaptation in the dark, vertical black and white stripes of a defined spatial frequency were presented to the animal. These stripes were rotated alternately clockwise and anticlockwise for 4 min. Mice were video tracked (EthoVision 6.1, Noldus), and the number of head movements was determined.

### Appendix A.10. Open Field

The open field test is used to measure voluntary locomotion activities. Single animals were placed in 60 cm × 60 cm empty arenas in a dimmed room, and video tracked (EthoVision 6.1, Noldus) for 20 min. For each animal, the time spent at the center and periphery of the arenas was recorded.

### Appendix A.11. Y Maze

The Y maze test was used to assess exploration. The system is composed of 3 opaque arms (35 cm length × 5 cm width × 10 cm eight) connected at 120° from each other. Mice were placed into a start arm and video tracked (EthoVision 6.1, Noldus) for 5 min to determine the number of entries into different arms.

### Appendix A.12 Elevated Plus Maze

The elevated plus maze test was used to assess anxiety. Mice were placed in an elevated plus maze consisting of two opposing open arms (exposed places) and two opposing closed arms (safer places) at 90° of each other. Mice were video tracked (EthoVision 6.1, Noldus) for 5 min to determine the time spent in each arm and the browsed distance.

### Appendix A.13 Contextual Fear-Conditioning Test

Fear (freezing behavior) was tested in a system consisting of a square box (25 × 25 × 25 cm) with black walls containing an electrifiable steel grid floor placed on a pressure plate (connected to a software), a sound and a luminous source (Bioseb). On the first day of the assay (training), mice were placed in the box for 2 min for habituation. Five consecutive times separated by 30 s recovery times, a mild foot shock was administrated during 2 s. On the second day of the assay, mice were placed in the same context for 4 min, and their freezing behavior was recorded. Mean values were used for analyses.

### Appendix A.14. Morris Water Maze

The Morris water maze was used to test spatial learning and memory. In the first phase of the test (spatial learning retention), mice were placed to swim in a circular pool with a diameter of 113 cm filled with water at 26 °C. The pool was virtually divided in four quadrants (northeast (NE), southeast (SE), southwest (SW) and northwest (NW)) with several visual cues around the pool, and a platform was placed at the NE quadrant. After a first day of training, the time latency to reach the platform and the time spent in each quadrant were measured for 3 consecutive days. In the second phase of the test (unlearning), mice were placed in the water maze from which the platform had been removed. The time spent in the NE target quadrant was measured over 1 min of swimming time for 4 consecutive days.
