# Supplementary material for: In Vitro and In Vivo Characterization of MCT1 Inhibitor AZD3965 Confirms Preclinical Safety Compatible with Breast Cancer Treatment
Source: Cancers (Basel). 2021 Feb 2;13(3):569. doi: 10.3390/cancers13030569 (PMC7867268; doi:10.3390/cancers13030569)

In vitro and in vivo characterization of MCT1 inhibitor AZD3965 confirms preclinical safety compatible with breast cancer treatment

--- Supplementary data ---

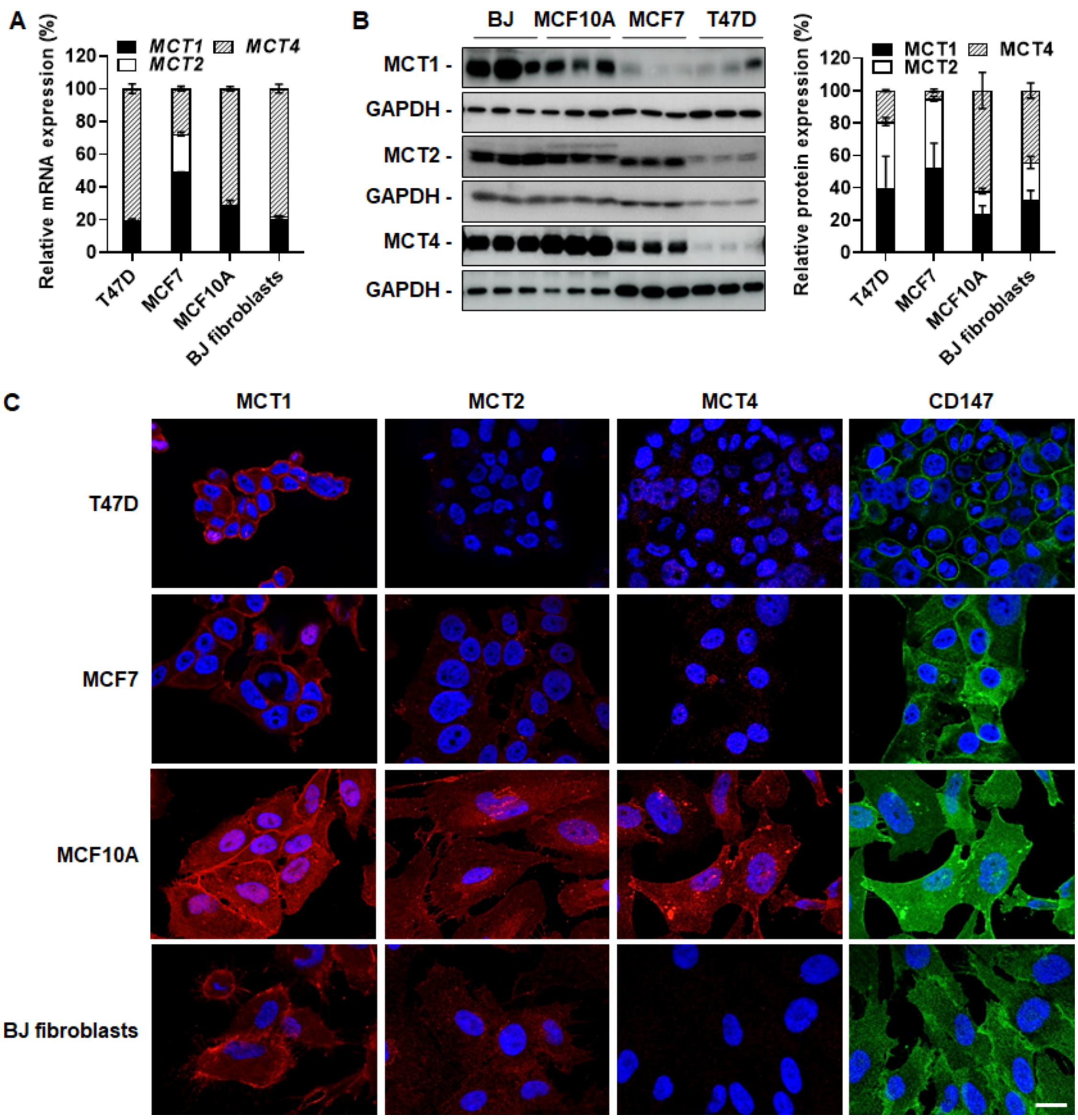

Figure S1. MCTs and CD147/basigin expression in breast-associated cells. (**A-C**) T47D, MCF7, MCF10A cells and BJ fibroblasts were assayed in medium containing 25 mmol/L glucose with 10 mmol/L GlutaMAX, 10% FBS and without added lactate. (**A**) Relative basal mRNA expression of *MCT1*, *MCT2* and *MCT4*. Cumulated expression served for normalization (100%) (n = 3). (**B**) Relative basal protein expression of MCT1, MCT2 and MCT4. Representative western blots are shown with GAPDH as a loading control. Cumulated expression served for normalization (100%) (n = 3). (**C**) Representative pictures of immunocytochemical staining of MCT1 (*red*), MCT2 (*red*), MCT4 (*red*) and CD147/basigin (*green*) on T47D, MCF7, MCF10A cells and BJ fibroblasts. Cell nuclei are stained in blue with DAPI. Bar = 20 µm. All data are show as means ± SEM.

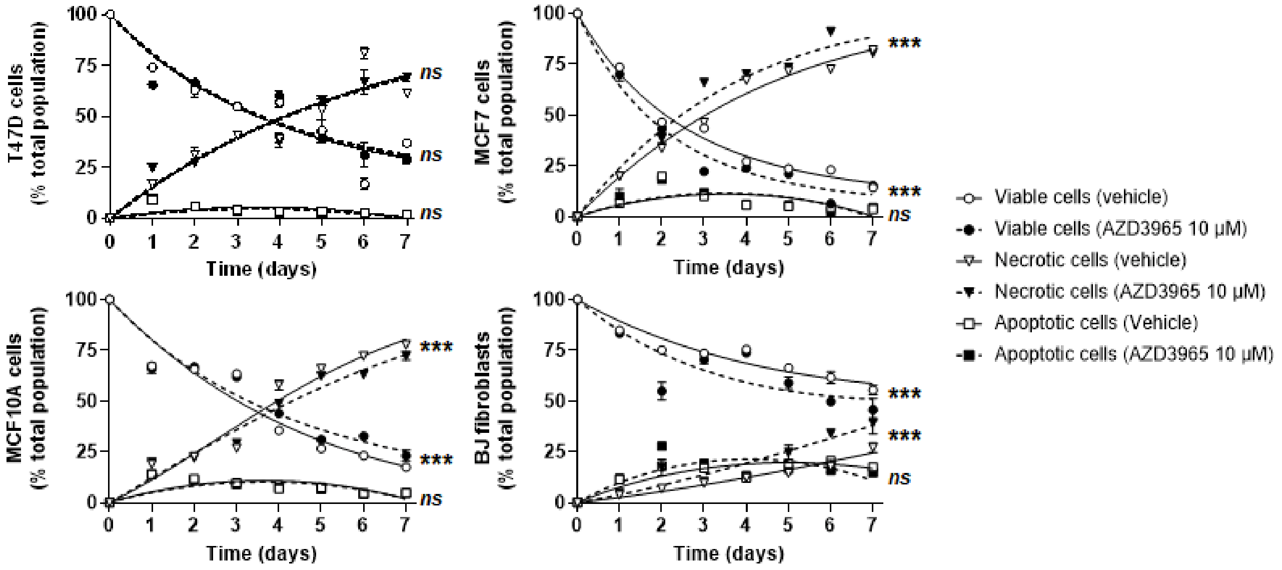

Figure S2. Long-term culture with lactate as only exogenous resource induces breast-associated cell necrosis with limited impact of additional MCT1 inhibition by AZD3965. T47D, MCF7, MCF10A cells and BJ fibroblast density was assayed in medium containing 10 mmol/L sodium *L*-lactate, no glucose, no glutamine, and 1 % FBS. On day 0, cells were treated ± 10 µmol/L of AZD3965. Graphs show the percentage of viable cells, necrotic cells and apoptotic cells over time determined using flow cytometry after Annexin V and propidium iodide labeling (n = 4-6). All data are show as means ± SEM. *** $P < 0.05$, *ns* $P > 0.05$ comparing whole curves; by two-way ANOVA.

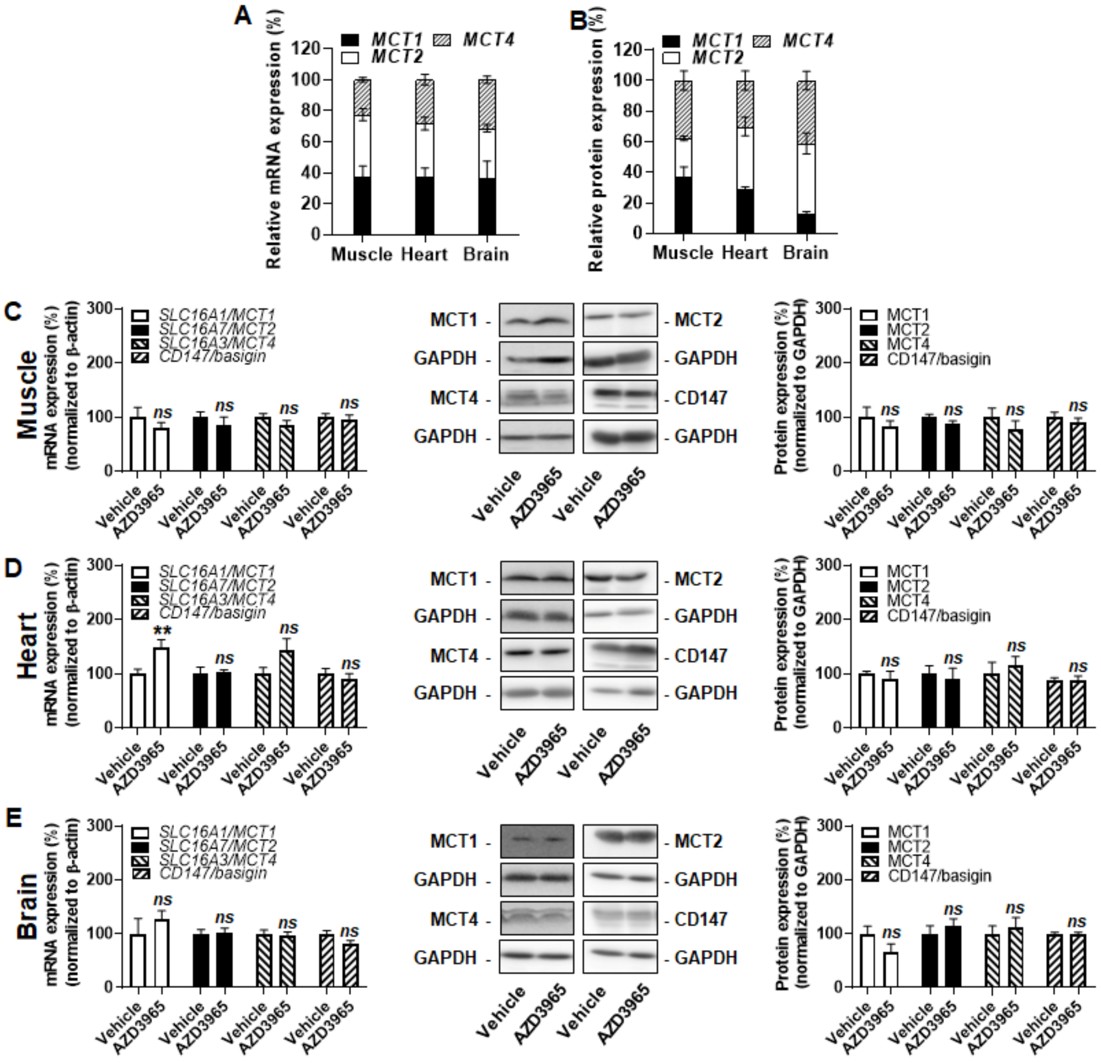

Figure S3. A chronic treatment with AZD3965 does not alter the expression of MCTs and CD147/basigin in mouse skeletal muscles, heart and brain. (**A-E**) Mouse tissues were collected on the day of sacrifice of Group 2 depicted in **Figure 7A**. (**A**) Relative mRNA expression of *MCT1*, *MCT2* and *MCT4* in the muscles, heart and brain of vehicle-treated mice. Cumulated expression served for normalization (100%) (n = 4-6). (**B**) Relative protein expression of MCT1, MCT2 and MCT4 in the muscles, heart and brain of vehicle-treated mice. Representative western blots are shown with GAPDH as a loading control. Cumulated expression served for normalization (100%) (n = 4-6). (**C**) mRNA (*left panel*) and protein (*middle and right panels*) expression of MCT1, MCT2, MCT4 and CD147/basigin in the gastrocnemius muscles of mice treated ± 100 mg/Kg AZD3965 (n = 4-24 for RT-qPCR, n = 5-6 for WB). (**D**) As in (C) but in whole mouse hearts (n = 3-6 for RT-qPCR, n = 5-6 for WB). (**E**) As in (C) but in whole mouse brains (n = 5-6 for RT-qPCR, n = 5-6 for WB). All data are show as means ± SEM. ** $P < 0.01$, *ns* $P > 0.05$ compared to corresponding tissues from vehicle-treated animals; by Student's t test (C-E).

Figure S4. Uncropped western blots

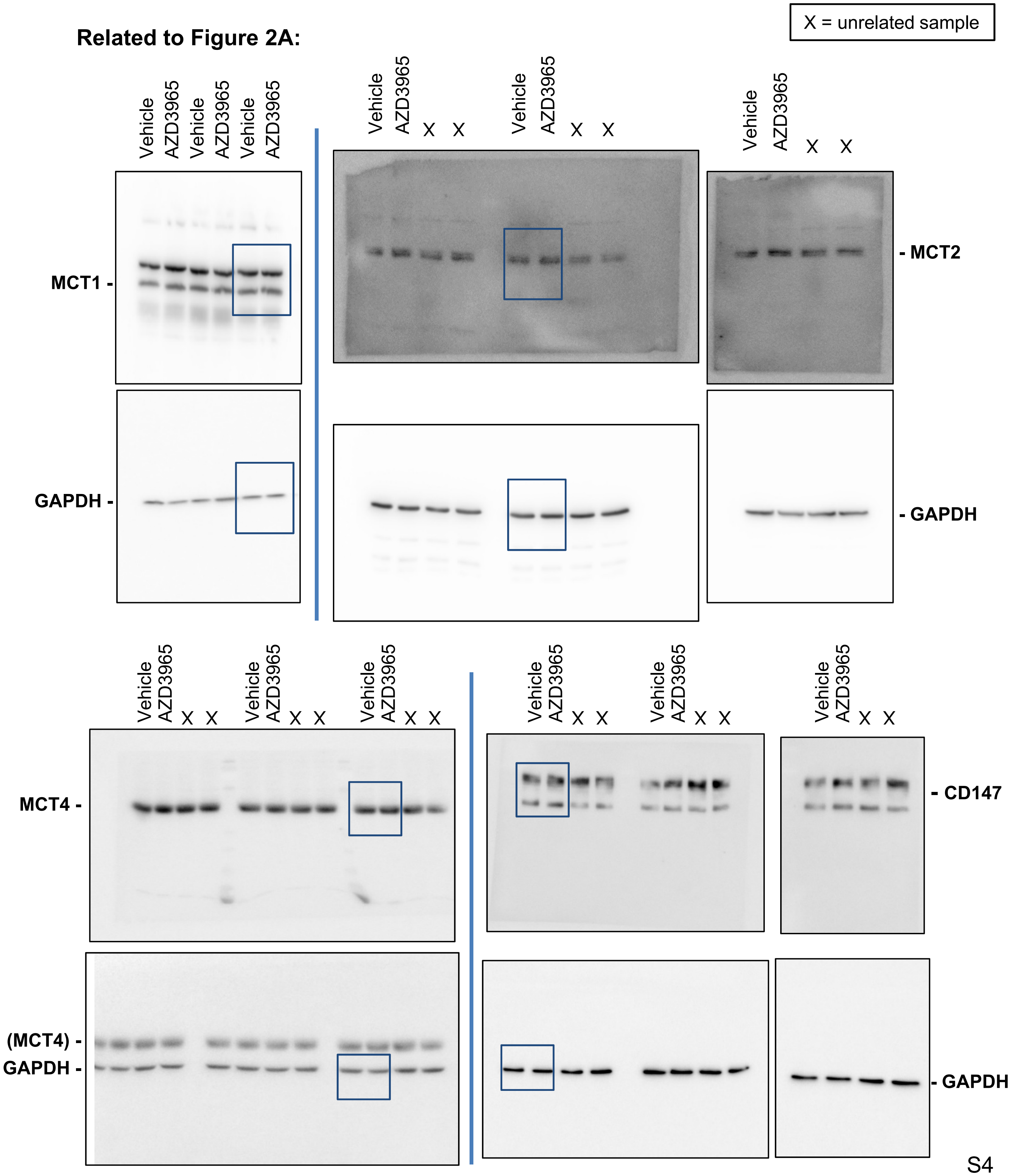

Related to Figure 2B:

X = unrelated sample

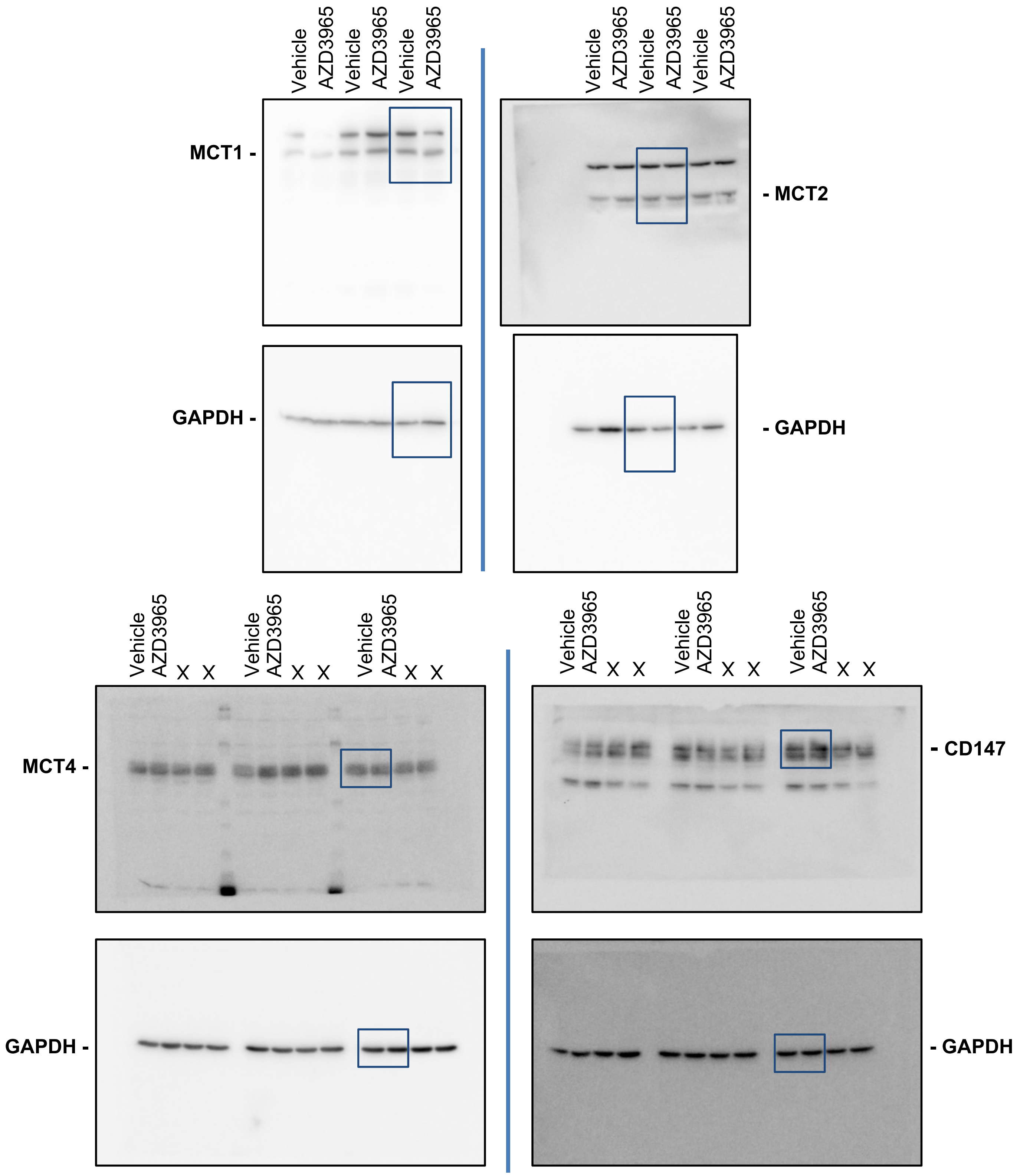

Related to Figure 2C:

X = unrelated sample

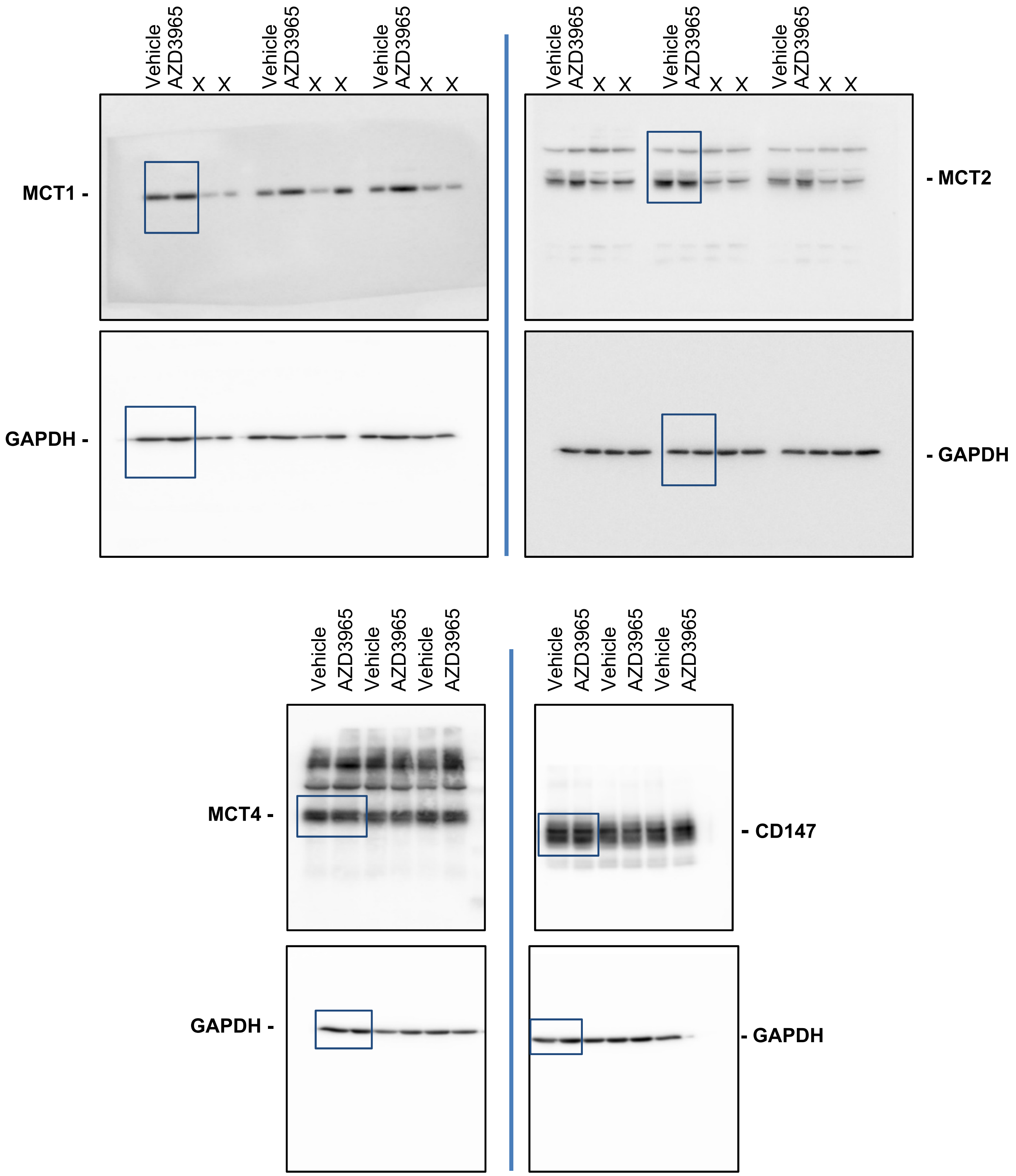

Related to Figure 2D:

X = unrelated sample

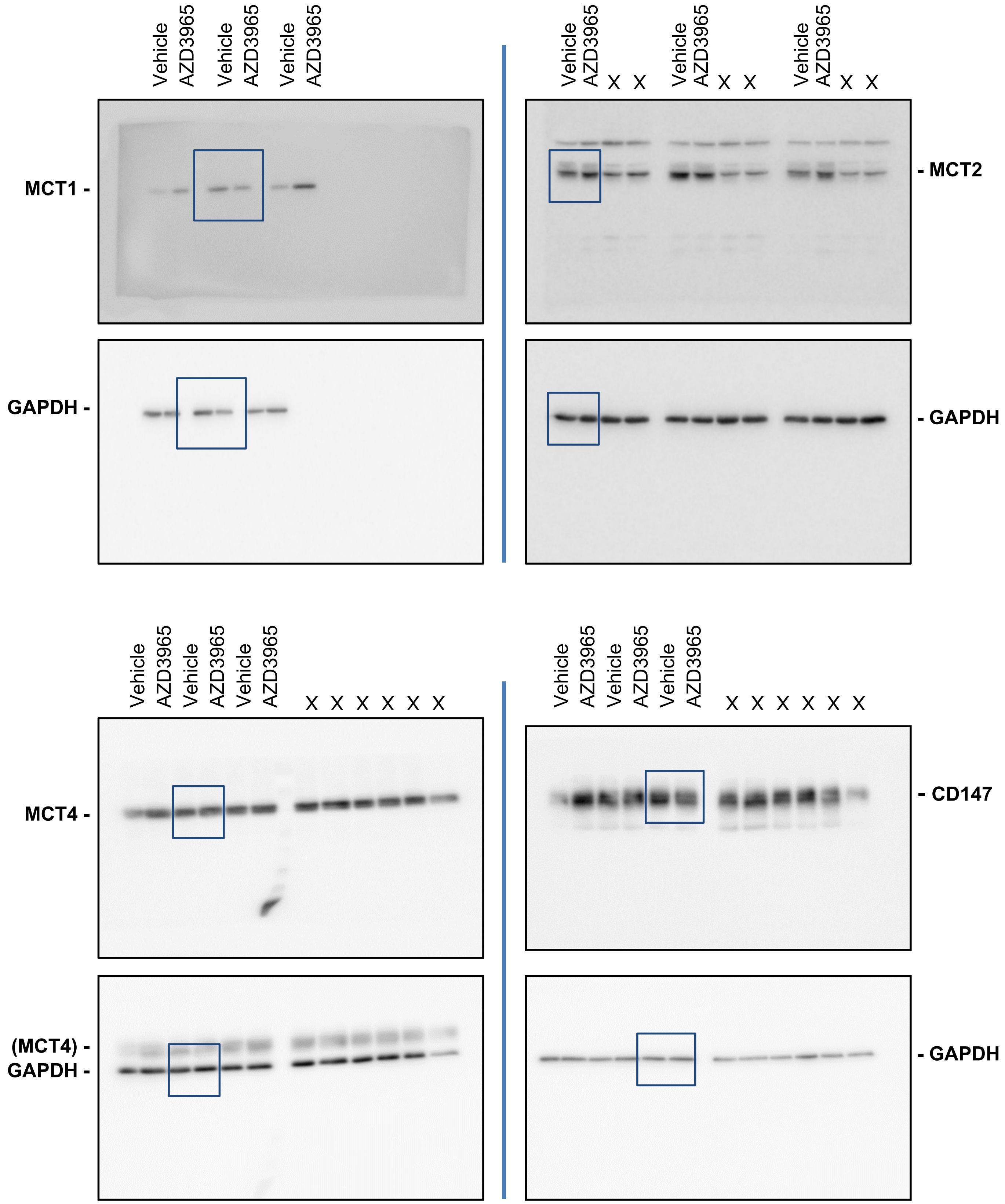

Related to Figure 5A:

X = unrelated sample

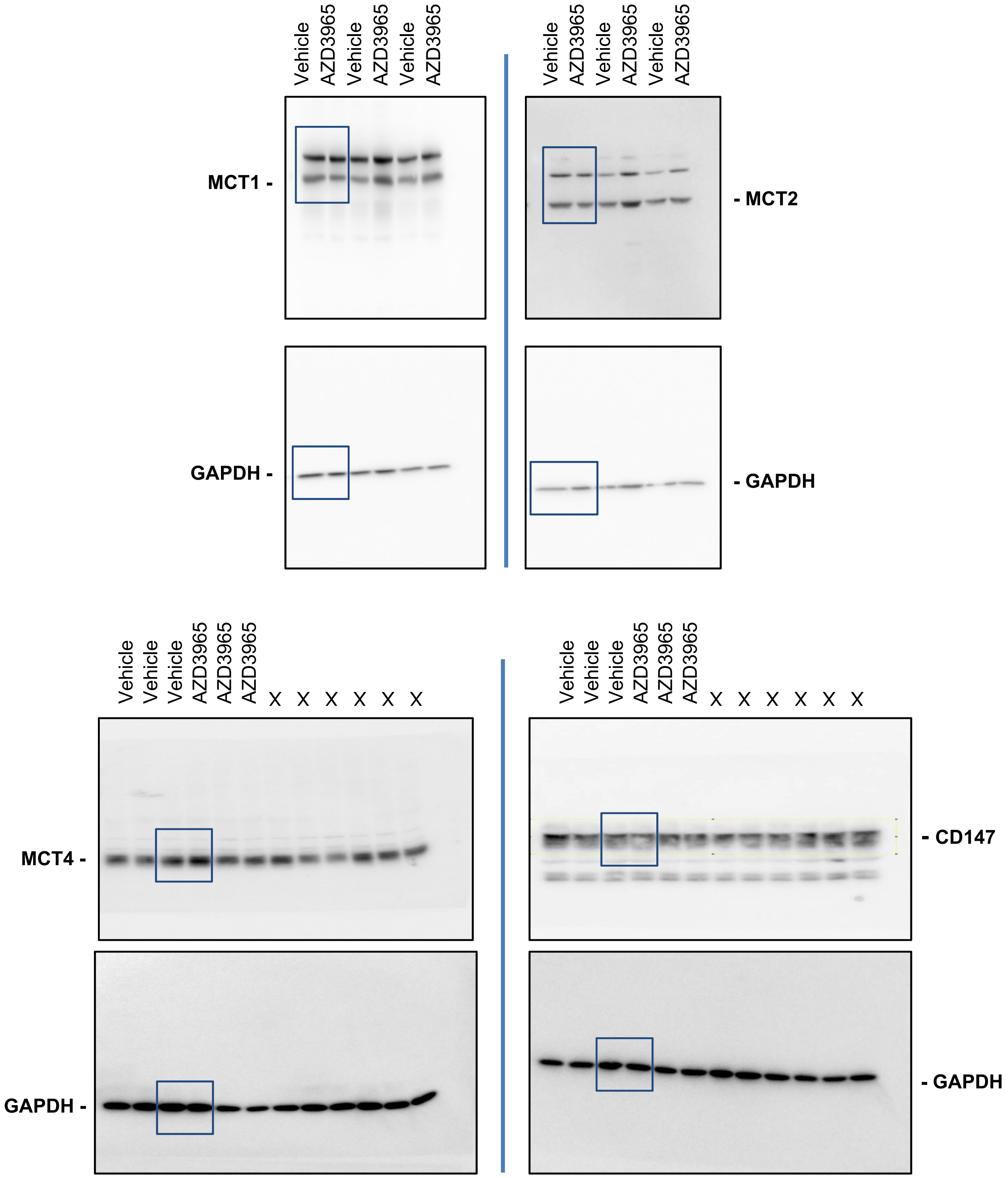

Related to Figure 5B:

X = unrelated sample

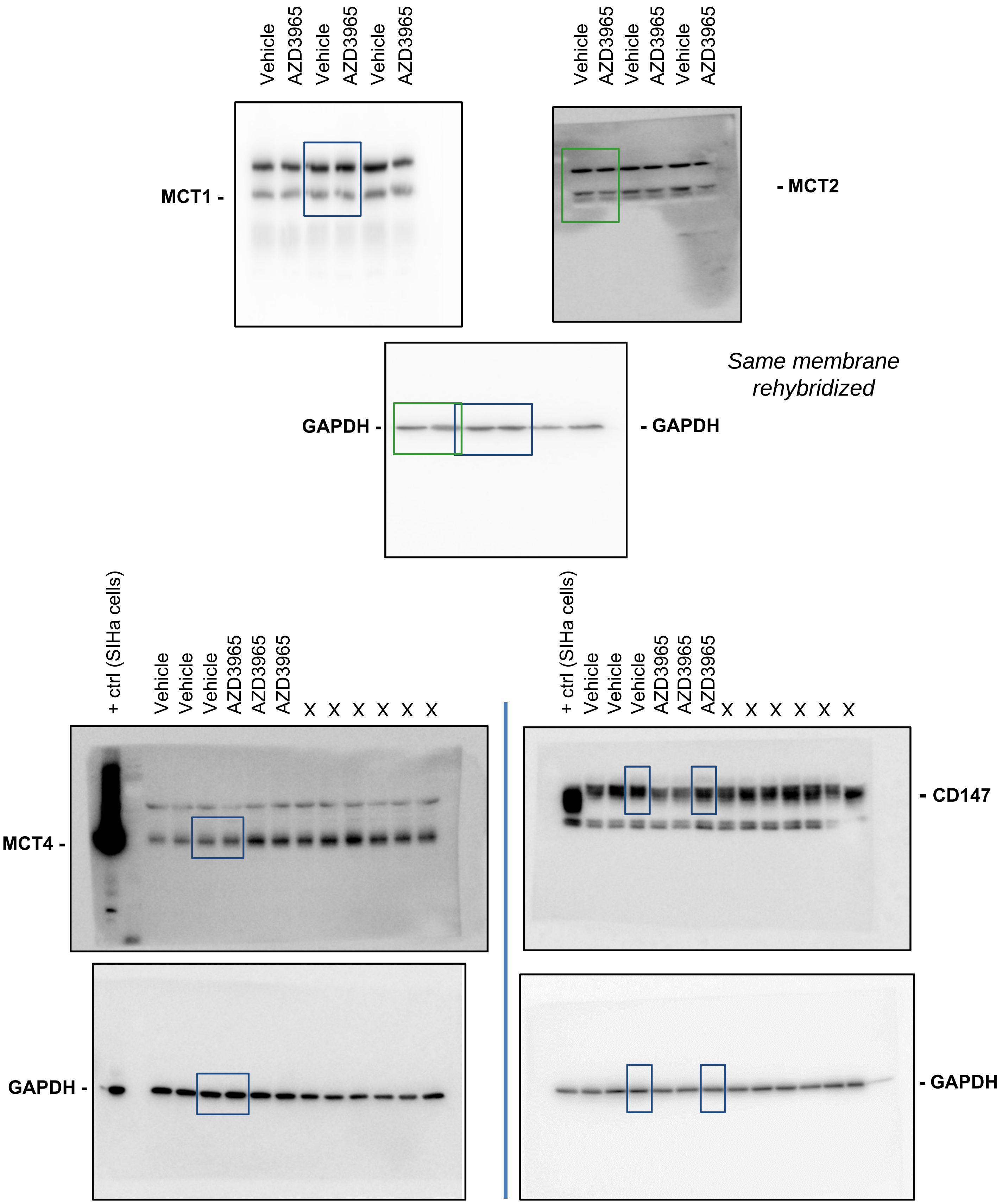

Related to Figure 5C:

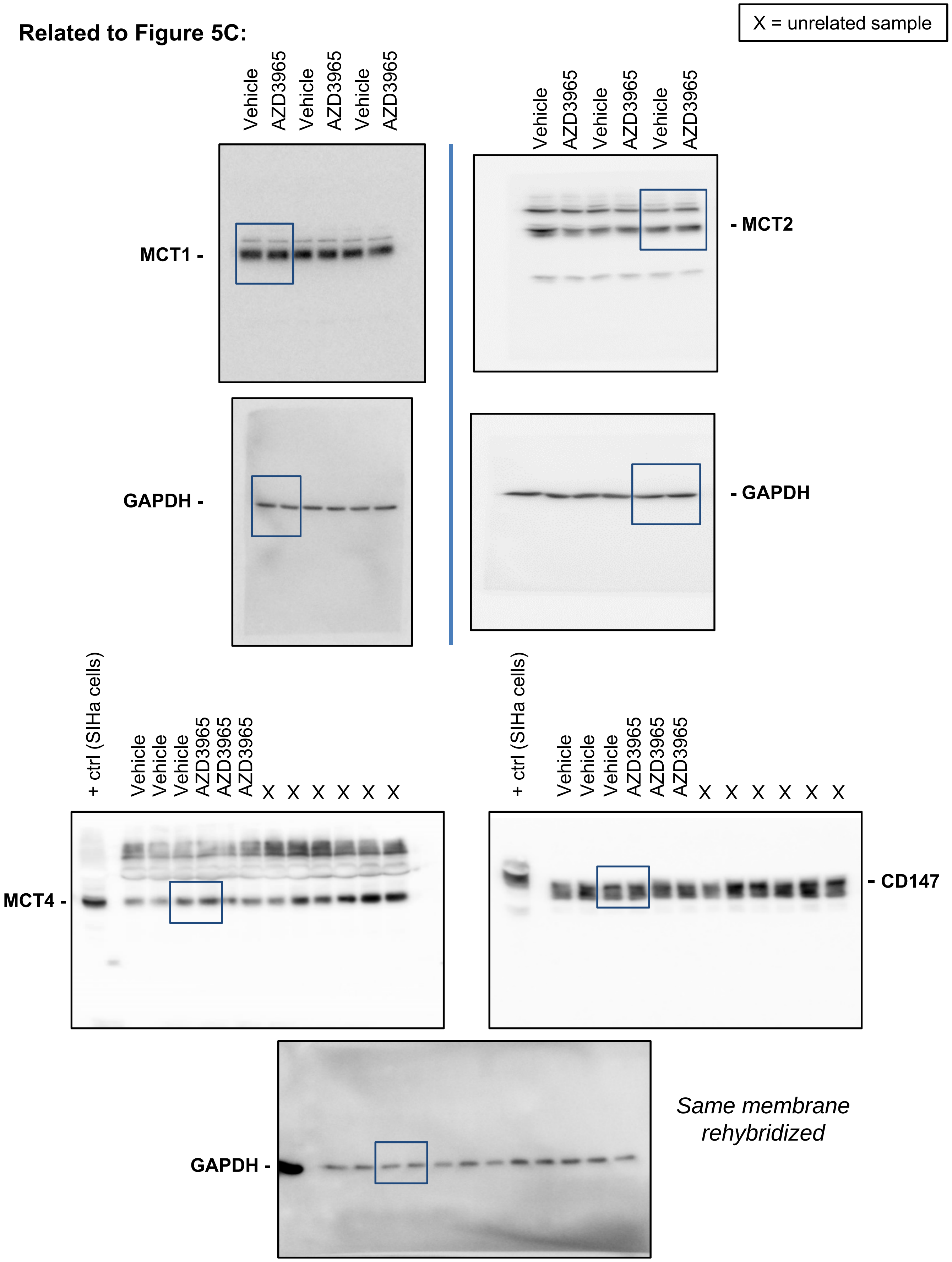

Related to Figure 5D:

X = unrelated sample

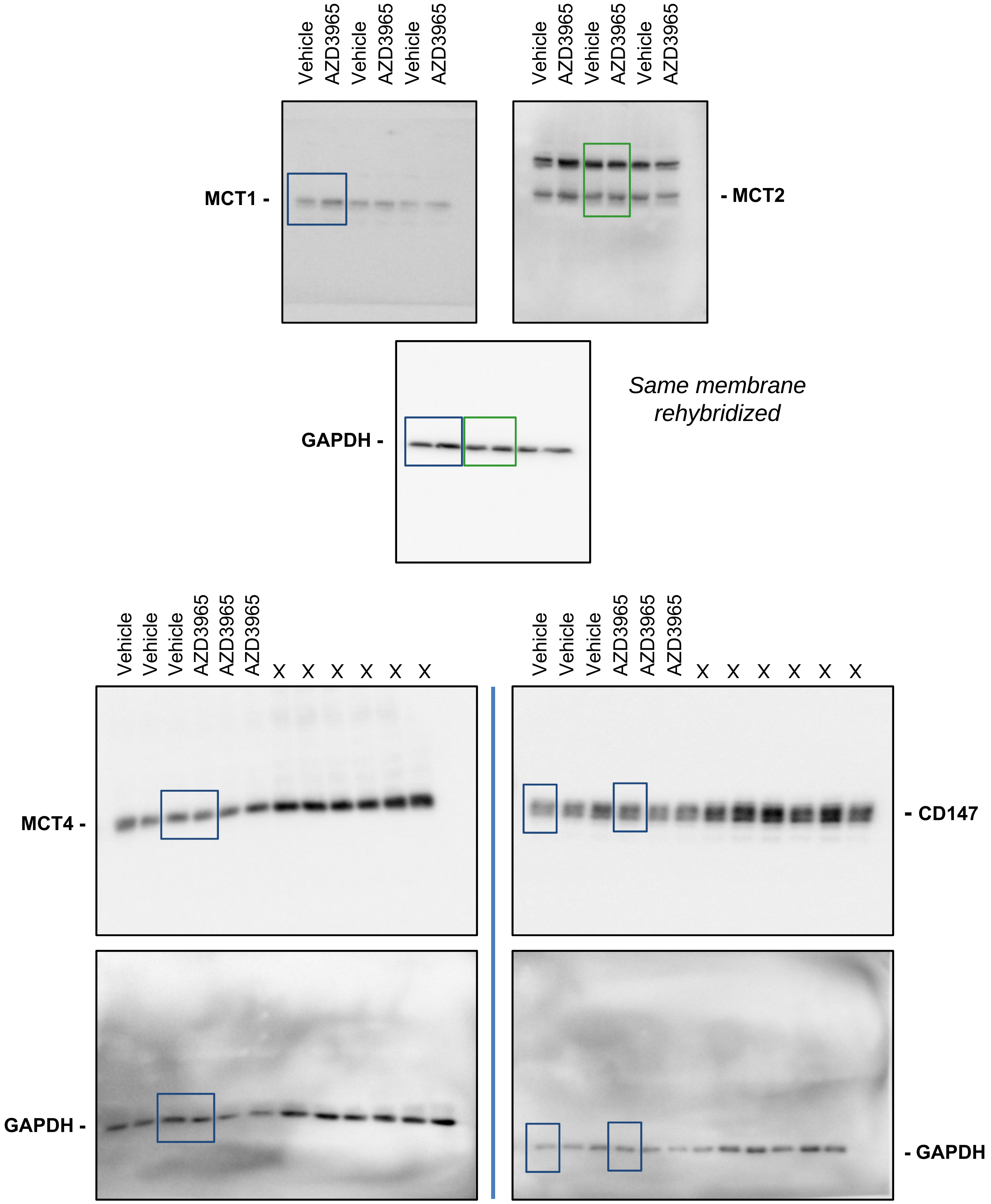

Related to Figure S1B:

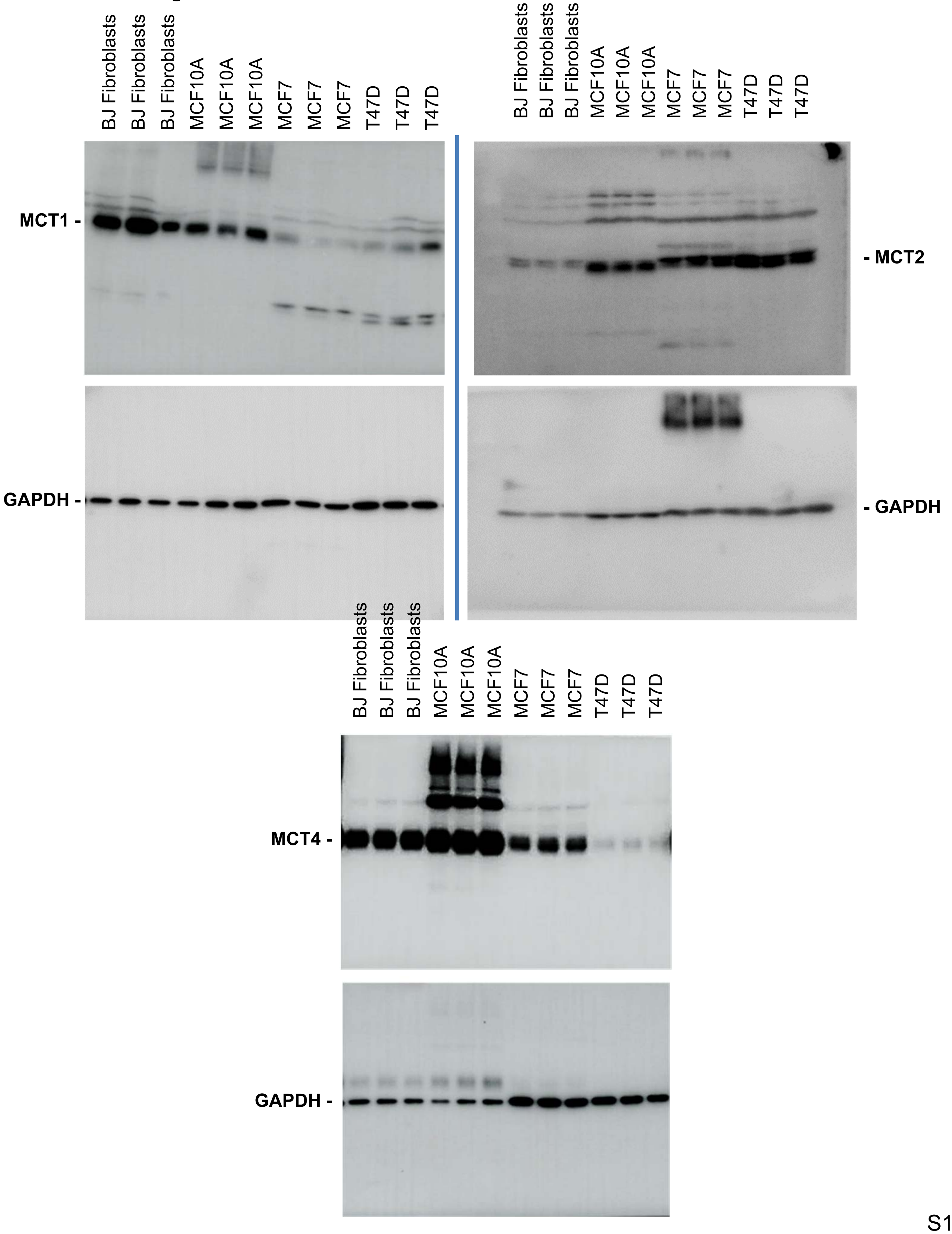

Related to Figure S2C:

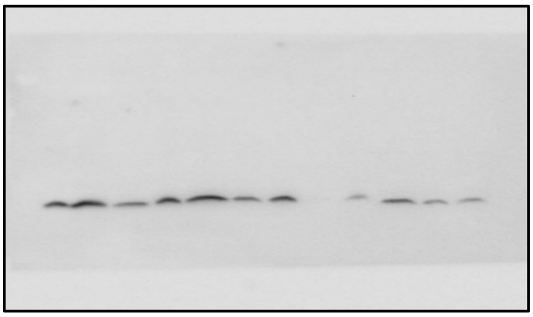

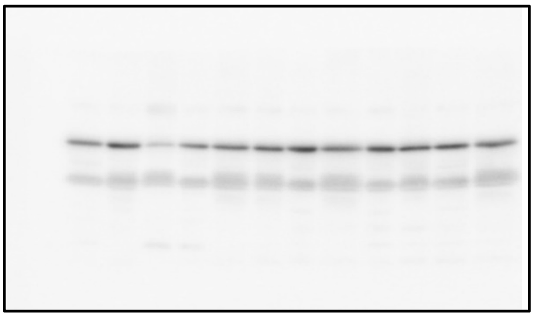

Supplement: Supplementary file 1 [file cancers-13-00569-s001.pdf]
